# Taxonomic quasi‐primes: peptides charting lineage‐specific adaptations and disease‐relevant loci

**DOI:** 10.1002/pro.70241

**Published:** 2025-08-25

**Authors:** Eleftherios Bochalis, Michail Patsakis, Nikol Chantzi, Ioannis Mouratidis, Dionysios V. Chartoumpekis, Ilias Georgakopoulos‐Soares

**Affiliations:** ^1^ Institute for Personalized Medicine, Department of Biochemistry and Molecular Biology The Pennsylvania State University College of Medicine Hershey Pennsylvania USA; ^2^ Department of Internal Medicine, Division of Endocrinology Medical School, University of Patras Patras Greece; ^3^ Huck Institute of the Life Sciences Pennsylvania State University University Park Pennsylvania USA

**Keywords:** evolution, K‐mers, reference proteomes, taxonomic quasi‐primes, taxonomies, tree of life

## Abstract

The identification of succinct, universal fingerprints that enable the characterization of individual taxonomies can reveal insights into trait development. Here, we introduce taxonomic quasi‐primes, peptide k‐mer sequences that are exclusively present in a specific taxonomy and absent from all others. By analyzing 24,073 reference proteomes, we identified these unique peptides at the superkingdom, kingdom, and phylum ranks. These sequences exhibit remarkable uniqueness at six‐ and seven‐amino‐acid lengths. For instance, the seven‐mer SAPNYCY is found in 98.11% of eukaryotic species, while being completely absent from archaeal, bacterial, and viral reference proteomes. Functional analysis demonstrated that taxonomic quasi‐prime containing proteins are enriched for processes defining a lineage, such as synaptic signaling in Chordata. Structural analysis revealed that these peptides are preferentially located within proteins, participating directly in enzymatic active sites, mediating protein–protein interactions, and stabilizing ligand binding. Moreover, we show that in human proteins, highly conserved Chordata quasi‐prime loci are 2.08‐fold more likely to harbor pathogenic variants than surrounding regions, directly linking these evolutionary signatures to disease. This study establishes taxonomic quasi‐primes as markers that illuminate evolutionary pathways and provide a powerful method for identifying functionally indispensable and disease‐relevant loci, which warrant further therapeutic and diagnostic investigation.

## 1 INTRODUCTION

The number of available reference proteomes has rapidly increased in recent years, a trend that is expected to continue in the foreseeable future (UniProt Consortium, 2023). The availability of a large and diverse set of proteomes provides an opportunity to increase our understanding of protein sequence and functional diversity in nature across taxonomic ranks. Such research could reveal insights into trait development through findings pertaining to mechanisms of sequence conservation and divergence, the emergence of proteins with novel functional roles, and can have applications in biomarker discovery and pathogen surveillance (Al‐Amrani et al., 2021; Lacerda & Reardon, 2009). These advances can be facilitated if the availability of ever‐expanding proteomic information is coupled with novel and insightful algorithms to process this abundance of biological information.

Peptide k‐mers are defined as oligopeptide sequences of length k and are often used in proteomics analyses (Moeckel et al., 2024). The number of possible peptide k‐mers exponentiates with k‐mer length, leading to oligopeptide sequence uniqueness even at low k‐mer lengths (Georgakopoulos‐Soares et al., 2021; Mouratidis et al., 2024). Because of their ease of identification, peptide k‐mers have numerous applications, including mass‐spectrometry‐based proteomics (Chapman, 2013), motif search and evolutionary studies (Wen et al., 2014), taxonomic classification, antimicrobial resistance and pathogen detection (ValizadehAslani et al., 2020), and the identification of therapeutic targets (Hajisharifi et al., 2014; Wu et al., 2019) among others.

**FIGURE 1:**
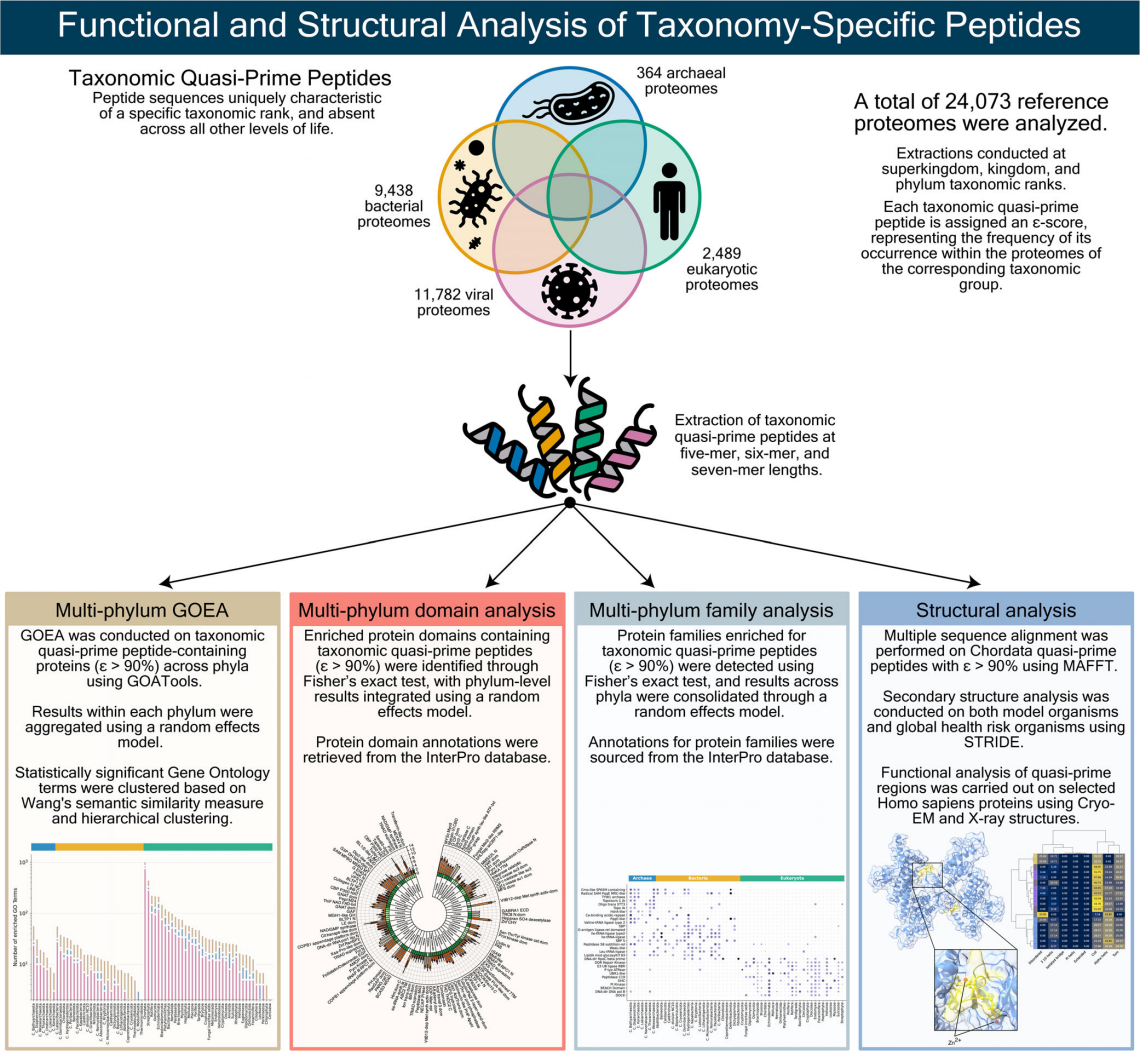
Overview of taxonomic quasi‐prime peptide identification and analysis pipeline. Identification of taxonomic quasi‐prime peptides was performed for 24,073 reference proteomes at the superkingdom, kingdom, and phylum ranks. To understand the roles of taxonomic quasi‐prime peptides, a thorough characterization was performed, including Gene Ontology enrichment analyses, protein domain and family analyses, and structural examinations.

We recently described the concept of quasi‐prime peptides, which are the shortest peptides that are unique to a species's proteome and are absent from every other known proteome (Mouratidis et al., 2023). Here, we have extended the concept of quasi‐prime peptides to incorporate taxonomic ranks, identifying k‐mer peptides that are present in one or multiple species of a taxonomic rank, but absent from every known proteome outside that rank. Using 24,073 reference proteomes, we provide proof of the existence of k‐mer peptides with this property at the superkingdom, kingdom, and phylum ranks (Figure 1). We demonstrate the role of taxonomic quasi‐prime peptides in evolutionary divergence and taxonomic adaptations and provide evidence that Chordata quasi‐prime loci are more likely to harbor pathogenic variants in human proteins.

## 2 MATERIALS AND METHODS

### 2.1 Proteomic datasets

Reference proteomes were obtained from UniProt (Release 2024_01), comprising a total of 24,073 species, including 364 Archaeal, 9438 Bacterial, 2489 Eukaryotic, and 11,782 viral proteomes.

Peptide k‐mer extraction was performed as previously described in Mouratidis et al. (2023), for k‐mer lengths of five to seven amino acids. We defined T as the superset of all considered taxonomies, K as a given k‐mer and P as a proteome. K‐mer lengths below five amino acids were not considered, as tetrapeptides are highly prevalent and in the human proteome all possible tetrapeptides are observed (Chantzi et al., 2024; Georgakopoulos‐Soares et al., 2021). Octapeptides and longer oligopeptides were not considered as the possible proteome space becomes extremely large (Wen et al., 2014), limiting the set of k‐mers that are shared between multiple species in a taxonomy.

*Definition of k‐mer*: We say that a k‐mer K belongs to a taxonomic rank $T_{i}$ if and only if there exists at least one species S in $T_{i}$ such that K is found in S, that is:

(1) $$K\in T_{i}\;\text{if and only if}\;\exists S\in T_{i}:K\in S$$

*Definition of taxonomy*: For species classification, a taxonomy organizes species into hierarchical categories such as Superkingdoms, Kingdoms, and Phyla.

*Definition of ε‐score*: The ε‐score for a k‐mer K in a taxonomic rank $T_{i}$ is a measure of the frequency with which K appears in the proteomes of taxonomic rank $T_{i}$.

(2) $$\varepsilon \left(K,T_{i}\right)=\frac{\left|\left\{P\in T_{i}:K\in P\right\}\right|}{\left|T_{i}\right|}\times 100,\varepsilon \left(K,T_{i}\right)=0,\ldots ,100$$

An ε‐score equal to zero indicates complete absence of the k‐mer K across all species of the taxonomic rank $T_{i}$, while an ε‐score of one hundred indicates universal presence of k‐mer K across all member species of the taxonomic rank $T_{i}$.

*Definition of taxonomic quasi‐prime peptides*: Taxonomic quasi‐prime peptides were defined as peptide sequences present in species of a taxonomic rank $T_{i}$ and absent from all species outside that taxonomic rank.

(3) $$Q\left(T_{i}\right)=\left\{K|K\in T_{i}\wedge \forall j\neq i:K\notin T_{j}\right\}$$

### 2.2 Taxonomic quasi‐prime peptide protein matching

The Peptide Match command line tool (Chen et al., 2013) was used to map taxonomic quasi‐prime peptides to the protein sequences containing them. The Lucene index needed for Peptide Match was created using UniProt reference proteome sequences containing SwissProt and TrEMBL entries. After the mapping process, a file containing the taxonomic quasi‐prime peptides of interest, the corresponding UniProt entry identifier, and the sequence coordinates containing the peptide was obtained.

### 2.3 Species clustering based on phylum ε‐scores

Uniform Manifold Approximation and Projection (UMAP; McInnes et al., 2018) was employed using the Python library umap‐learn (version 0.5.7) to analyze phylum taxonomic quasi‐prime seven‐mers corresponding to the top 50th percentile of ε‐scores within each phylum. This approach aimed to visualize how species clustered in two‐dimensional space based on their taxonomic quasi‐prime composition. A semi‐supervised, density‐based UMAP was implemented, incorporating a target weight of 0.25 for clustering based on labels. The algorithm parameters were configured with 30 neighbors and a minimum distance of 0.1 to optimize cluster resolution.

### 2.4 Multi‐species gene ontology (GO) enrichment analysis

Multi‐species GO enrichment analysis (GOEA) was performed on taxonomic quasi‐prime peptide‐containing proteins using GOATools (version 1.4.12) (Klopfenstein et al., 2018) at the phylum level. A GOEA (Ashburner et al., 2000) was performed for each species, and results were combined using appropriate statistical methods (see “Enrichment score combination across same phylum species” section). We selected taxonomic quasi‐prime peptides with an ε‐score above 90.00% for each phylum in Archaea, Bacteria, and Eukaryotes. The study population for each analysis consisted of the taxonomic quasi‐prime peptide‐containing proteins, whereas the background population was represented by all the proteins expressed from the corresponding species. The Open Biological and Biomedical Ontologies (OBO) 1.4 file (.obo), containing ontology information and needed for the GOATools package, was obtained from the Gene Ontology Resource (Gene Ontology Consortium et al., 2023) (https://geneontology.org/, release 2024‐09‐08) and included a total of 40,939 GO terms and 7,894,411 annotations for 5,426 species. Finally, the GO annotation file representing the relationship between UniProt entry and GO terms was downloaded from the Gene Ontology Annotation Database (version 222, released on August 05, 2024, https://ftp.ebi.ac.uk/pub/databases/GO/goa/UNIPROT/goa_uniprot_all.gaf.gz). Species were filtered to keep only those that possessed a protein count greater than 10 to ensure statistical robustness. Study population protein counts were not propagated up the GO graph during GOEA. *P*‐values were adjusted using the Benjamini–Hochberg method, and only GO terms with an adjusted *p*‐value less than 0.05 were selected, comprising the statistically significant GO terms.

### 2.5 GO result clustering

Statistically significant GO terms were clustered into broader representative terms to minimize redundancy and noise and highlight functional themes present across phyla. Each GO term class (biological process (BP), molecular function (MF), and cellular component (CC)) within each phylum was handled separately and Wang's semantic similarity measure (Wang et al., 2007) was used to calculate the pairwise similarity of terms. These similarity values were converted to a distance matrix for agglomerative hierarchical clustering with the average linkage method. The optimal clustering threshold specifically for each GO term class was set to 0.54 for BP, 0.535 for MF and 0.52 for CC (Bettembourg et al., 2015). Post‐clustering a representative term for each cluster was selected based on the adjusted p‐value and ties between terms were resolved using the presence percentage across species belonging to an individual phylum and the combined LOR, where terms with the highest value were characterized as representative. For the representative terms a weighted average LOR was calculated using the standard error as weight followed by winsorization at the 95th percentile to minimize the effect of extreme values. GO terms that were not assigned to any cluster were also retained, if the term's adjusted *p*‐value was less than 0.05.

### 2.6 Multi‐species protein entry enrichment analysis

Taxonomic quasi‐prime peptide‐containing proteins were subjected to multi‐species protein entry enrichment analysis. During the analysis, the presence of taxonomic quasi‐primes within functional protein domains and the protein family composition proteins containing them was analyzed. Protein entry data were obtained from the InterPro database (version 101.0 updated on July 25, 2024) (Paysan‐Lafosse et al., 2023), which contains 14,950 domain and 26,089 family entries. For the enrichment analysis, Fisher's exact test was implemented, from which an odds ratio (OR) and a *p*‐value was calculated for each entry. Haldane–Anscombe correction (Agresti, 1999) was applied to all cells of the 2 × 2 contingency table. Only entries with a *p*‐value less than 0.05 and an OR greater than 1.0, to analyze only enriched entries downstream, and with presence across more than 1 species were subjected to effect combination, whereas entries present only in one species were retained, if they passed the *p*‐value and OR cut‐off.

### 2.7 Enrichment score combination across same phylum species

Single species GOEA and protein entry enrichment analysis results were combined into a phylum representative unified score (one for each analysis). This was achieved using a meta‐analytic random effects model. The model involved calculating the natural logarithm of OR (LOR) and the corresponding initial fixed‐effect weights. Then using Cochran's Q and the subsequent Tau‐squared we calculated the final adjusted weights that also represent the between‐study variance. These weights were used to determine the phylum‐wide enrichment value for each item. The statistical significance of each enrichment score was assessed using a modified *z*‐score and *p*‐values derived from a *t*‐distribution. Multiple testing correction was implemented through the Benjamini–Hochberg procedure, and items with an adjusted *p*‐value greater than 0.05 were filtered out. More details about the mathematical procedures behind the random‐effects model can be found in the Supplementary Material.

### 2.8 Secondary and tertiary structure analysis

The secondary structures of taxonomic quasi‐prime peptides from key model and global health risk organisms were analyzed. Protein PDB files were obtained from the AlphaFold Protein Structure Database (Jumper et al., 2021; Varadi et al., 2024) updated as of September 2024. For each protein, taxonomic quasi‐prime peptide regions were extracted and their secondary structure was identified using the STRIDE (STRuctural IDEntification) algorithm (Frishman & Argos, 1995) as provided by the ssbio (version 0.9.9) tool package. Peptides that presented with no hydrogen‐bonds were characterized to have a Disordered conformation. For the structural analysis, PDB files presenting the interaction between selected proteins and ligands were downloaded from the RCSB Protein Data Bank (Berman et al., 2000) (updated as of 2024, download timestamp: November 21, 2024). Only conformations obtained through Cryo‐EM or x‐ray were selected. Protein visualization and hydrogen‐bond detection was performed using UCSF ChimeraX (version 1.8) software (Meng et al., 2023).

### 2.9 Assessing the biochemical robustness of taxonomic quasi‐prime peptides

To address if the uniqueness of the taxonomic quasi‐prime peptides extended into biochemical properties other than k‐mer sequence, we performed substitution matrix analysis. We selected Chordata taxonomic quasi‐prime seven‐mers with an ε‐score exceeding 90.00% and for each peptide we generated all possible single‐amino acid variants that have a favorable score. We employed the BLOSUM62 (Henikoff & Henikoff, 1992) substitution matrix implemented through the blosum (version 2.0.3) Python package, where a peptide variant was classified as biologically conservative and was retained for further analysis, if the substitution had a positive score in the matrix. We considered substitutes only for canonical amino acids, excluding the ambiguity codes “X,” “*,” “B,” “Z,” and “J.” The set of biologically conservative peptides was then mapped against the proteins of all 24,073 reference proteomes.

### 2.10 Protein ortholog mapping and multiple sequence alignment

Protein orthologs for human proteins across the Chordata phylum were obtained through the EggNOG (version 5.0.0) (Huerta‐Cepas et al., 2019) database. The orthologous groups of choice were later subjected to multiple sequence alignment (MSA) using the MAFFT (version 7) command line tool (Katoh & Standley, 2013) with the options: ‐‐localpair ‐‐maxiterate 1000 ‐‐amino ‐‐thread 5. The resulting alignments were trimmed using ClipKIT (version 2.3.0) (Steenwyk et al., 2020) with the options ‐smart‐gap to remove poorly aligned protein regions and improve the phylogenetic signal by focusing on well‐conserved segments. The trimmed results were visualized using Jalview (version 2.11.4.1) (Waterhouse et al., 2009). Sequences were ordered based on pairwise similarity, and taxonomic quasi‐prime‐containing regions were highlighted whereas distant regions with no peptides of interest were hidden from the visualization.

### 2.11 Pathogenicity prediction in taxonomic quasi‐prime protein regions

To predict the pathogenicity of single‐nucleotide missense variants, we employed AlphaMissense (Cheng et al., 2023), (version 3, updated as of September 19, 2023, https://zenodo.org/records/10813168). The analysis included all possible missense variants (approximately 71 million) derived from 19,000 canonical protein‐coding transcripts in the human genome (hg38 build). Our study specifically focused on human proteins containing taxonomic quasi‐prime seven‐mers with ε‐scores exceeding 90.00%. We conducted a comparative analysis of the pathogenicity associated with missense mutations located within taxonomic quasi‐prime loci and those occurring outside these loci. Mutations with AlphaMissense scores below 0.1 were classified as likely benign, while those with scores above 0.9 were designated as highly pathogenic.

## 3 RESULTS

### 3.1 Derivation of superkingdom‐ and kingdom‐specific quasi‐prime peptides

**FIGURE 2:**
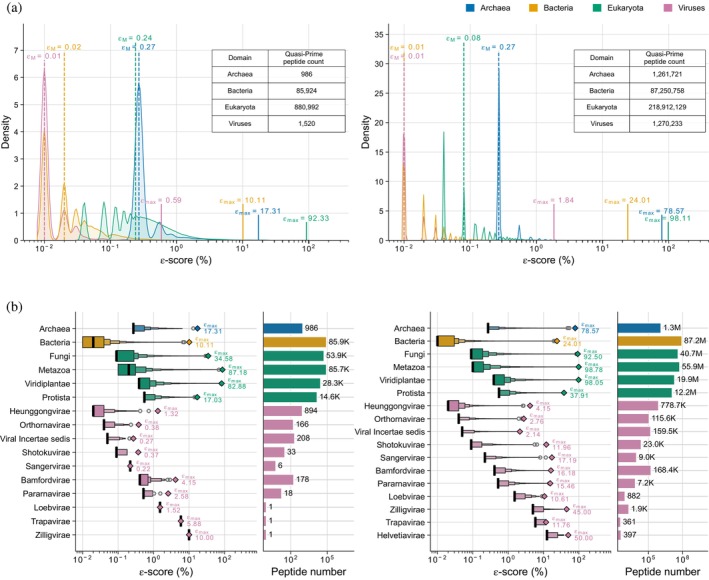
Taxonomic quasi‐prime peptide counts and ε‐score distribution. (a) Kernel density estimate plots showing taxonomic quasi‐prime peptide ε‐score distribution at superkingdom level. Dotted lines represent the median ε‐score (ε_M_) and solid lines represent the maximum ε‐score (ε_max_) value. Tables inside each plot display the number of unique taxonomic quasi‐prime peptide counts. (b) Taxonomic quasi‐prime peptide ε‐score distribution and peptide count at kingdom level. Left to right: Letter‐value plot of ε‐score distribution, where the ε_M_ value is depicted as a solid black line and the ε_max_ value as a rhombus. Bar plot of the unique taxonomic quasi‐prime peptide counts. (Data for taxonomic quasi‐prime six‐mers are displayed to the left and seven‐mers are displayed to the right).

Taxonomic quasi‐prime five‐mers are identifiable at the superkingdom level and are exclusively found within the Eukaryota. We identified 12 distinct five‐mers with ε‐scores ranging from 0.52% to 3.74%, and a median ε‐score (ε_M_) of 1.67% (Supplementary Figure 1). The sequences of these 12 peptides are detailed in Supplementary Table 1. At the six‐mer level, we find that the median percentage of species containing these six amino acid peptides is 0.01% in viruses, 0.02% in Bacteria, 0.24% in Eukaryotes, and 0.27% in Archaea (Figure 2a.). Similar patterns are observed for taxonomic quasi‐prime seven‐mers (Figure 2a). The larger number of taxonomic quasi‐primes identified in Eukaryotes stems from their larger proteome sizes (Spearman's correlation coefficient *ρ* = 0.963, *p*‐value <0.001) (Supplementary Figure S4). Additionally, due to the large k‐mer space, these findings translate to a considerable number of superkingdom‐specific peptides. Specifically, at k‐mer lengths six and seven, we observe thousands of peptides that are only found in species of a single superkingdom (Figure 2a). For instance, we observe 85,373 and 86,483,511 peptides found uniquely in bacterial proteomes at six and seven amino acids k‐mer lengths, respectively (Figure 2a).

The maximum ε‐score (ε_max_) of taxonomic quasi‐prime six‐mers was 0.59%, 10.10%, 17.31%, and 92.33% in viruses, Bacteria, Archaea, and Eukaryotes respectively. This means that the same taxonomic quasi‐prime six‐mer is found in 92.33% of reference Eukaryotes and is otherwise absent from all Bacteria, Archaea, and viruses. For taxonomic quasi‐prime seven‐mers, peptide SAPNYCY had an ε_max_ of 98.11% for eukaryotic species, which maps to proteins of the serine/threonine phosphatase family, which are highly conserved in Eukaryotes (Ohama, 2019) (Figure 2a, Supplementary Figure 3a).

Next, we analyzed the distribution of taxonomic quasi‐primes across various organismal and viral kingdoms, observing significant variability within individual groups. This was particularly pronounced in viral kingdoms, where ε_M_ values ranged from 0.02% to 10.00% at the six‐mer level and from 0.02% to 5.00% at the seven‐mer level in Heunggongvirae and Zilligvirae, respectively (Figure 2b). Among eukaryotic kingdoms, the lowest ε_M_ value was found in Fungi (0.09%), while the highest was observed in Protista (0.55%) (Figure 2b). The ε_max_ of taxonomic quasi‐prime six‐mers ranged from 17.03% to 87.18%, while for seven‐mers, values ranged from 37.91% to 98.78% in Protista and Metazoa, respectively (Supplementary Table 2). For Metazoa, the taxonomic quasi‐prime seven‐mer with the highest ε‐score was CKGFFKR, which mapped to proteins associated with the nuclear hormone receptor family. Proteins included in this family show strong sequence conservation and little evidence for positive selection in Metazoans (Krasowski et al., 2005) (Figure 2b, Supplementary Figure 3b).

### 3.2 Derivation of phylum‐specific quasi‐prime peptides

**FIGURE 3:**
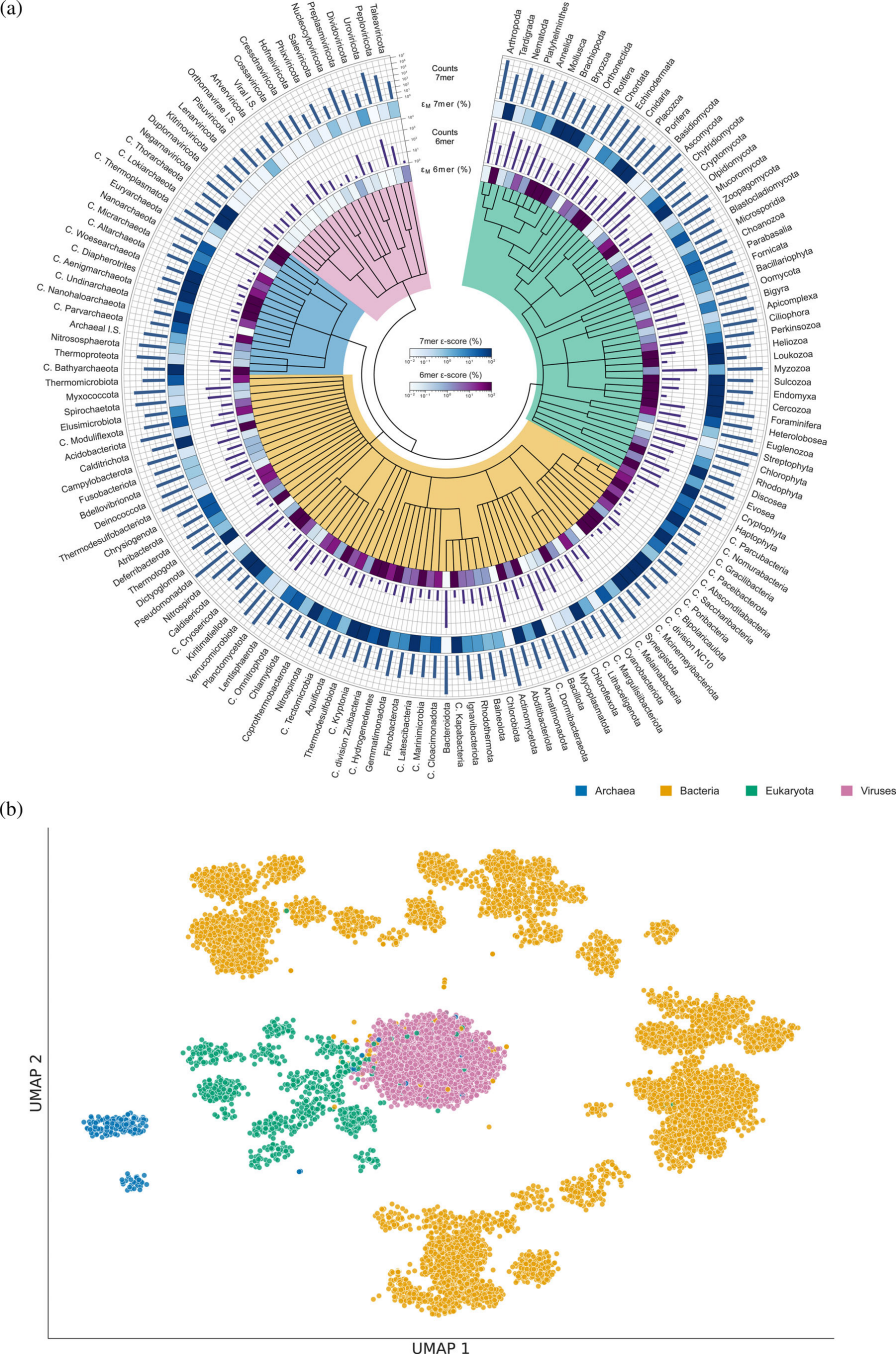
Taxonomic quasi‐prime peptide statistics at phylum level. (a) Circos plot depicting the taxonomic organization of superkingdoms, kingdoms, and phyla at the archaeal, bacterial, eukaryotic, and viral levels (center). The number of unique taxonomic quasi‐prime peptides, alongside the median ε‐score value (ε_M_) at six‐mer and seven‐mer lengths, is also shown. Inner to outer: ε_M_ for six‐mer taxonomic quasi‐prime peptides; total six‐mer taxonomic quasi‐prime peptide counts; ε_M_ for seven‐mer taxonomic quasi‐prime peptides; total seven‐mer taxonomic quasi‐prime peptide counts. (b) UMAP plot depicting the clustering of reference proteomes based on their taxonomic quasi‐prime seven‐mers. Only the top 50th percentile of taxonomic quasi‐primes based on their ε‐score was used for the clustering. C, Candidatus; I.S, incertae sedis.

We investigated the presence and distribution of taxonomic quasi‐primes across various phyla. At the six‐amino‐acid sequence length, ε_M_ values displayed substantial variation. In eukaryotic phyla, values ranged from 0.14% in Ascomycota to 100.00% in Foraminifera and 16 other eukaryotic phyla. For bacterial phyla, ε_M_ values spanned from 0.03% in Pseudomonadota to 100.00% in Abditibacteriota and 17 additional bacterial phyla. Archaeal phyla exhibited values between 0.43% in Euryarchaeota and 100.00% in seven candidate archaeal phyla. In viral phyla, ε_M_ values ranged from 0.02% in Uroviricota to 10.00% in Taleaviricota (Figure 3; Supplementary Figures 4 and 5). A similar distribution pattern was observed for the seven‐amino‐acid sequence length. In eukaryotic phyla, Ascomycota again exhibited the lowest ε_M_ value at 0.14%, while Porifera reached the maximum value of 100.00%, shared with 16 other eukaryotic phyla. Among bacterial phyla, ε_M_ values ranged from 0.03% for Pseudomonadota to 100.00% for Abditibacteriota and 18 additional bacterial phyla. For archaeal phyla, Euryarchaeota showed the lowest ε_M_ value of 0.43%, while Candidatus Bathyarchaeota and seven other candidate archaeal phyla attained ε_M_ of 100.00%. Viral phyla exhibited values ranging from 0.02% in Uroviricota to 12.50% in Dividoviricota (Figure 3; Supplementary Figures 4 and 5). We also observed that superkingdoms cluster by taxonomic quasi‐primes (Figure 3b). The complete distribution of ε_M_ values alongside ε_max_ for each phylum is available at the Supplementary File 1.

Across all three cellular superkingdoms, several phyla exhibited an ε_max_ of 100.00%, whereas for viral phyla the ε_max_ was capped at 50.00%. This is likely the result of viruses' rapid evolution in response to host immune pressures resulting in viral phyla encompassing greater genetic variation than cellular phyla. For both Streptophyta and Nematoda, we analyzed the proteins from which these highly phylum‐specific peptide seven‐mers originated. In Streptophyta, the taxonomic quasi‐primes TPWPGNN, EHFCIHA, and THHEYIQ were identified with an ε_max_ of 99.11%. These peptides were found in *Arabidopsis thaliana* within the proteins Cellulose Synthase A Catalytic Subunit 4 (UDP‐forming), 3‐Ketoacyl‐CoA Synthase 1, and Callose Synthase 5, respectively (Supplementary Figure 5c–e). In Nematoda, a single peptide, ICPKYCA, was identified with an ε_max_ of 97.65%. This peptide is located in the cuticle collagen DPY‐13 protein of *Caenorhabditis elegans* (Supplementary Figure 5f), which is crucial for cuticle formation, serving both as an exoskeleton and a protective barrier against environmental challenges.

### 3.3 Taxonomic quasi‐primes enable the detection of loci divergence and functional adaptations across taxa

**FIGURE 4:**
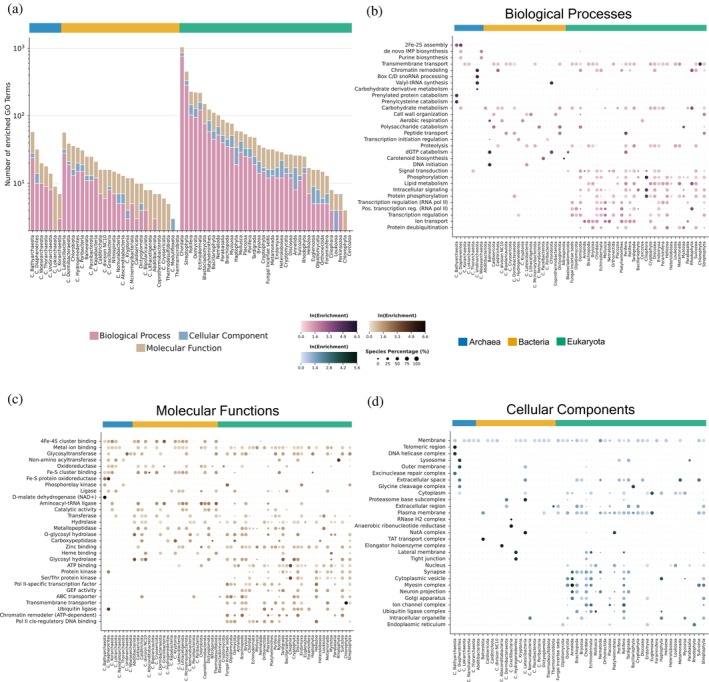
Multi‐phylum gene ontology (GO) enrichment analysis at the superkingdom level. (a) Stacked bar chart illustrating the total number of enriched GO terms, categorized by GO class, at the superkingdom level. (b)–(d) Heatmaps highlighting the top 10 GO class specific terms most broadly enriched across phyla within each of the three cellular organism superkingdoms. Dot size indicates the prevalence of each GO term among species within a given phylum, while dot color denotes the combined natural log (ln) of enrichment values, reflecting the strength of enrichment. Heatmaps are organized as follows: (b) biological processes, (c) molecular functions, (d) cellular components.

To explore the functional roles of taxonomic quasi‐prime peptides and their associated proteins, we conducted a GOEA tailored to individual taxa. As expected, eukaryotic phyla, such as Chordata and Streptophyta, exhibited the highest number of statistically significant (adjusted *p*‐value <0.05) enriched GO terms, while Archaea and Bacteria showed smaller comparable numbers. This finding in Eukaryotes is consistent with their larger proteomes and developmental complexity (Figure 4a).

**FIGURE 5:**
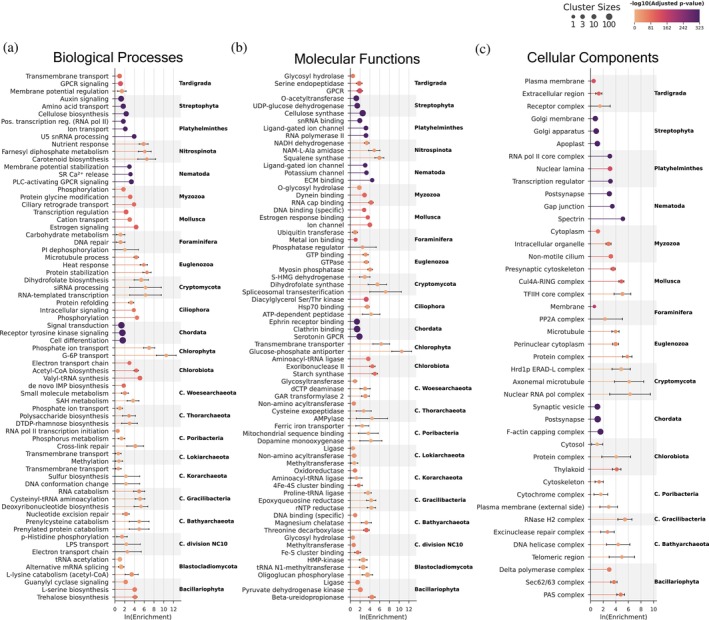
Gene ontology (GO) term cluster enrichment analysis across superkingdom representative phyla, with taxonomic quasi‐prime peptides. Lollipop plots display the mean enrichment of the top three most enriched clustered GO terms for representative phyla of the cellular organism superkingdoms, categorized into: (a) Biological processes, (b) molecular functions, (c) cellular components. Only GO terms with an adjusted *p*‐value less than 0.05 and a species representation greater than 5% are included. Dot size represents cluster size, lollipop color represents the −log10 adjusted *p*‐value, and error bars show the 95% confidence interval of the calculations. GO terms have been grouped into broader clusters using Wang's semantic similarity measure combined with hierarchical clustering.

The results of the GOEA correctly pinpointed GO terms in major phyla that are fundamental to their known biology. In Chordata, we observed significant enrichment for terms such as ion transport, receptor tyrosine kinase signaling, synaptic vesicle, and postsynapse, all of which are hallmarks of the complex nervous system that characterizes this phylum (Figures 4b, 5a–c). Expanding on this track, we correctly identified ephrin receptor binding, which is known to be associated with nervous system development (Kao & Kania, 2011; Kullander et al., 2001) and angiogenesis (Cheng et al., 2002), and clathrin binding, known for clathrin‐mediated endocytosis (McMahon & Boucrot, 2011), to be highly associated with taxonomic quasi‐prime peptides (Figure 5b).

Our validating results are not limited to Chordata. For instance, in Mollusca, estrogen signaling and estrogen response binding are correctly found enriched, confirming their reliance on environmental uptake of estrogen hormones (Balbi et al., 2019) (Figure 5a, b). The enrichment of taxonomic quasi‐primes in the microtubules and axonemal microtubules of Euglenozoa and Cryptomycota, respectively, aligns perfectly with the critical role these components play in flagellar motility and host interactions (Jones et al., 2011). In archaeal and bacterial phyla, 4Fe‐4S cluster binding was found enriched.

Beyond this validation, our analysis also revealed more nuanced and novel insights. Membrane and transmembrane transport were universally enriched, highlighting how this fundamental process is fine‐tuned through lineage‐specific adaptations (Figure 4b, c). More strikingly, we identified unexpected enrichment of eukaryotic‐like functions in Poribacteria, a bacterial phylum known to be a symbiot of Porifera. The enrichment of GO terms related to the RNA polymerase II transcription initiation BP and the dopamine monooxygenase MF (Figure 5a, b) suggests that the known mimicry of eukaryotic proteins by this bacterium (Kamke et al., 2014) extends down to the level of its unique taxonomic quasi‐primes, a novel insight provided by our methodology.

### 3.4 Taxonomic quasi‐primes detect superkingdom‐ and phylum‐level protein adaptations

**FIGURE 6:**
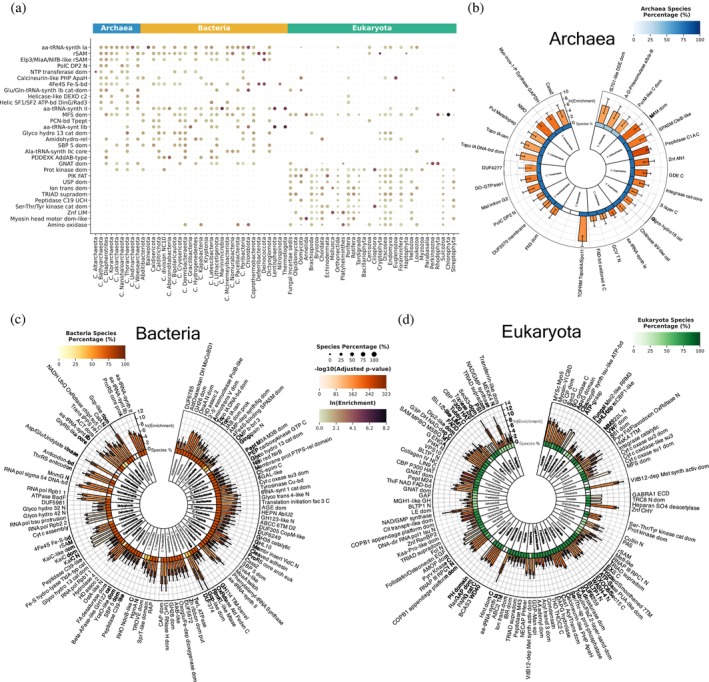
Preferential presence of taxonomic quasi‐primes in specific protein domains. (a) Size‐ and color‐coded heatmap displaying the top 10 protein domains with taxonomic quasi‐primes enriched across most phyla within each of the three cellular organism superkingdoms. Dot size indicates the prevalence of each protein domain within species of the respective phylum, while dot color corresponds to the combined ln(Enrichment) value for each domain. (b)–(d) Circos plots of the top three enriched protein domains per phylum with an adjusted *p*‐value less than 0.05 and a species prevalence greater than 5% for each cellular organism superkingdom. (b) Archaea, (c) Bacteria, and (d) Eukaryotes. Inner to outer: Phylum name; Heatmap depicting the percentage of the species within the respective phylum that have the specific protein domain enriched; Barplot showing the ln(Enrichment) value with error bars representing the 95% confidence interval of the calculation.

To investigate taxon‐specific functional changes, we analyzed the distribution of taxonomic quasi‐primes within protein domains and families, collectively termed as protein entries. Across all three superkingdoms, the major facilitator superfamily domain (MFS_dom), critical for transmembrane transport (Pao et al., 1998), emerged as a highly enriched domain (Figure 6a), underscoring its pivotal role in transport processes essential for cellular survival and adaptation (Complete protein domain names can be found at: Supplementary [Link], [Link]). This aligns with previously found enriched transmembrane transport BP shared across all three superkingdoms.

**FIGURE 7:**
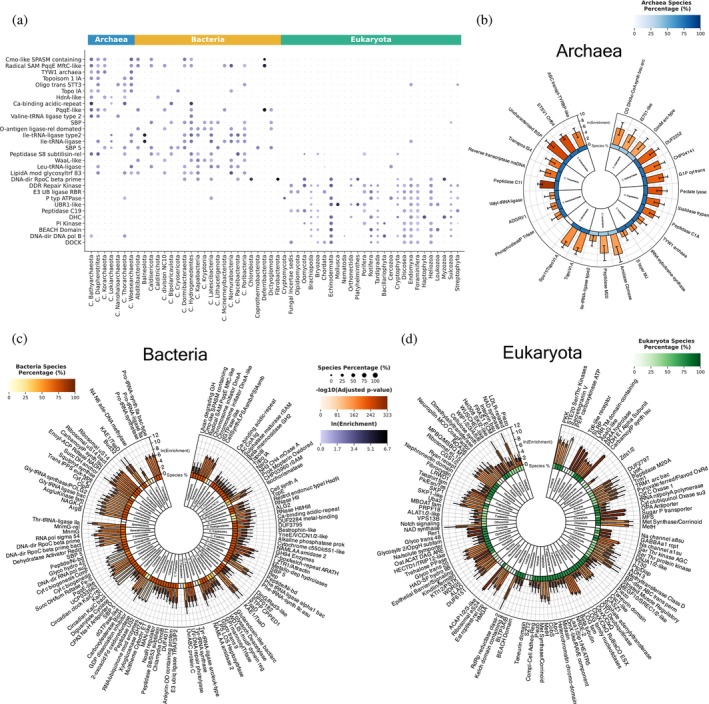
Analysis of the predominant taxonomic quasi‐primes in superkingdom specific protein families. (a) Size‐ and color‐coded heatmap identifying the top 10 enriched protein families widely present across phyla within each of the three cellular organism superkingdoms. Dot size indicates the prevalence of each protein family within species of the respective phylum, while dot color corresponds to the combined ln(Enrichment) value for each family. (b)–(d) Circos plots showcasing the top three most enriched protein families per phylum with an adjusted *p*‐value less than 0.05 and a species prevalence greater than 5% in: (b) Archaea, (c) Bacteria, and (d) Eukaryotes. Inner to outer: Phylum name; Heatmap depicting the percentage of the species within the respective phylum that have the specific protein family enriched; Barplot showing the ln(Enrichment) value with error bars representing the 95% confidence interval of the calculation.

Archaea and Bacteria displayed similar enrichment patterns in protein entries, which were largely absent in Eukaryota, and vice versa (Figures 6a and 7a). This indicates evolutionary conservation of protein entries across these two superkingdoms, due to their shared environmental challenges, horizontal gene transfer events, and adaptive responses. Enriched domains included those associated with oxidoreductase activity, transferase activity, catalytic activity, and metal ion binding. Particularly, the radical S‐adenosyl‐L‐methionine (rSAM) domain and its associated families (e.g., Elp3/MiaA/NifB‐like, PqqE‐like) (Figures 6a and 7a) were enriched, reflecting their roles in essential enzymatic functions. These families belong to the rSAM enzyme superfamily (Frey et al., 2008) and involve 4Fe‐4S cluster binding (4Fe4S_Fe‐S‐bd), critical for catalysis.

Aminoacyl‐tRNA synthetase domains were enriched in Archaea and Bacteria (aa‐tRNA‐synth Ia and aa‐tRNA‐synth II), with bacterial‐specific enrichment of the class II G/P/S/T subtype (Figure 6a). Three class Ia tRNA ligase protein families (Valine‐, Leucine and Isoleucine‐tRNA ligases) are found enriched predominantly in most archaeal and bacterial phyla (Figure 7a), supporting protein synthesis under extreme conditions. These adaptations compensate for the limited diversity of post‐transcriptional modifications in prokaryotes.

In Eukaryotes, enriched entries included the ion transport domain (Figure 6a), found in sodium, potassium, and calcium ion channels, the TRIAD supradomain present in the E3 ubiquitin ligase RBR family, and the myosin head motor domain alongside the dynein heavy chain family (Figure 7a). E3 ubiquitin ligases are highly conserved across Eukaryotes since they are a part of the ubiquitin‐proteasome system, involved in protein degradation and the maintenance of cellular homeostasis (Yang et al., 2021). Enrichment of motor protein entries highlights the necessity of quasi‐prime adaptations for intracellular transport and cellular motility.

We also identified the top three enriched entries within specific phyla. In Cyanobacteria, entries related to the circadian clock oscillator protein family (e.g., KaiC) (Figures 6b and 7b) are enriched, suggesting the high evolutionary conservation of taxonomic quasi‐primes in the regulation of day‐night cycles (Markson & O'Shea, 2009). In Candidatus Paceibacterota, the UV‐induced DNA damage repair photolyase family was enriched, warranting further study (Figure 7b). In Chordata, the heparan sulfate‐N‐deacetylase protein domain, critical for the biosynthesis of heparan sulfate (Sarrazin et al., 2011), is highlighted (Figure 6c). Also, the sodium channel A8 and A1 subunit families, alongside the gamma‐aminobutyric‐acid A receptor, alpha 1 subunit, are present across most Chordata species, further facilitating the involvement of taxonomic quasi‐primes in the function of the nervous system. Leucine‐rich repeat families were prominent in Arthropoda (Figure 7c) underscoring roles in immune defense and development, with emphasis on Toll‐like receptors (Dey et al., 2022) and extracellular matrix integrity (Matsushima et al., 2021). Little is known about how these families function in Arthropods, and the existence of highly specific taxonomic quasi‐primes emphasizes the need for further study.

### 3.5 Secondary structure analysis of taxonomic quasi‐primes in diverse organisms

We next analyzed the secondary structures of protein loci containing taxonomic quasi‐prime seven‐mers with an ε‐score greater than 90.00% across 13 model organisms and 17 pathogens of global health concern. Our findings revealed a distinct enrichment of taxonomic quasi‐primes in protein coils, followed by alpha helices and turns (Supplementary Figure 6). We evaluated the statistical significance of this structural preference using Kruskal–Wallis tests. For coiled‐like conformations (encompassing alpha helices, coils, and turns), the analysis yielded an H‐statistic of 213,788.82 with a *p*‐value <0.001 in model organisms, and an H‐statistic of 1352.19 with a *p*‐value <0.001 in pathogen proteomes. Nevertheless, we report significant differences depending on the organism studied; for instance, *Schizosaccharomyces pombe* shows a preference for taxonomic quasi‐primes in alpha helices and *Plasmodium falciparum* in disordered secondary structures (Supplementary Figure 6).

### 3.6 Multiple Sequence Alignment (MSA) and structural insights into chordata proteins

To further investigate the significance of highly conserved taxonomic quasi‐prime peptides in molecular function, we performed tertiary analysis on four representative Chordata proteins. These proteins were selected because they are prime representatives of the enriched Chordata GO terms and protein entries identified in the previous analyses. Also, these proteins contained taxonomic quasi‐prime seven‐mers with an ε‐score greater than 90.00% (Supplementary Table 3).

**FIGURE 8:**
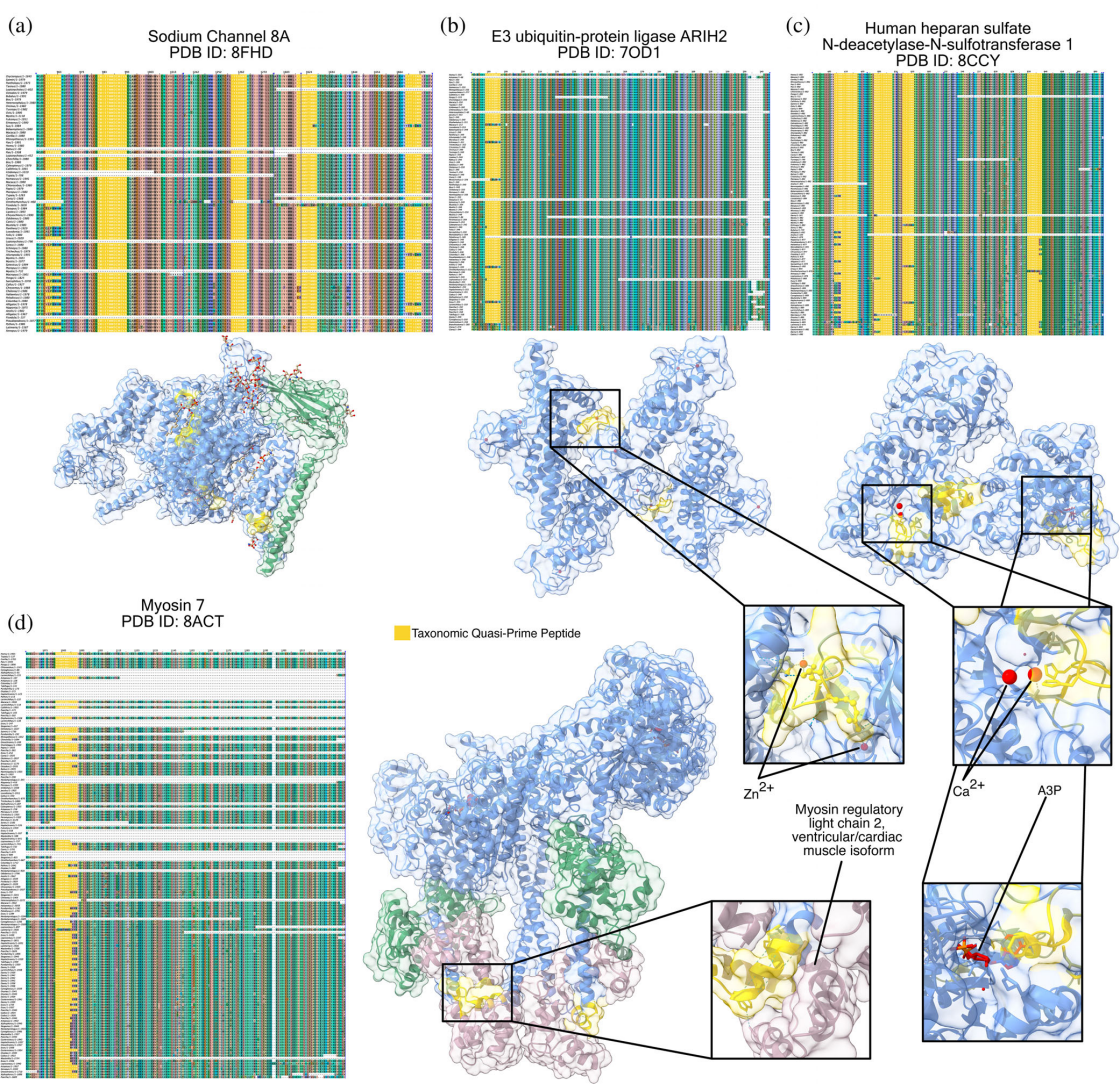
Structural profiling of taxonomic quasi‐primes across Chordata representative proteins. Multiple sequence alignment and structural representations of proteins containing taxonomic quasi‐primes with ε‐score greater than 90% in: (a) Human sodium channel 8A (*SCN8A*). (b) Human E3 ubiquitin‐protein ligase ARIH2 (*ARIH2*). (c) Human heparan sulfate N‐deacetylase‐N‐sulfotransferase 1 (*NDST1*). (d) Human myosin 7 (*MYH7*). Taxonomic quasi‐prime peptide region is displayed in yellow. Inset panels provide a detailed view of these regions, illustrating their specific locations within proteins, where they play a functional role. A3P: Adenosine‐3′‐5′‐Diphosphate.

The four selected proteins include:

- Sodium channel 8A (SCN8A) (Figure 8a): Represents the enriched ion transport BP (Figure 4b) and the sodium channel α8 family (Figure 7d).
- E3 ubiquitin‐protein ligase ARIH2 (ARIH2) (Figure 8b): Represents the enriched E3 ubiquitin ligase RBR family (Figure 7a) and its associated TRIAD supradomain (Figure 6a), which are linked to the enriched metal ion binding MF (Figure 4c).
- Heparan sulfate N‐deacetylase‐N‐sulfotransferase 1 (NDST1) (Figure 8c): Selected due to the enrichment of the heparan SO_4_ deacetylase domain (Figure 6d).
- Myosin 7 (MYH7) (Figure 8d): Represents the enriched myosin complex CC (Figure 4d) and the myosin head motor dom‐like domain (Figure 6a), which are fundamental for motor functions like muscle contraction.

Twelve taxonomic quasi‐primes, spread across various regions of the SCN8A protein, were identified (Figure 8a), suggesting a predominantly structural role rather than a functional one, as these regions are not known to actively interact with lipid substrates. In the ARI2 protein, three taxonomic quasi‐primes were found to interact directly with zinc ions through coordinate covalent bonds at the Cys257, Cys260, and His265 sites. These sites have been characterized as essential for the structural stability and catalytic activity of the enzyme (Figure 8b), since they are present in the IBR linker domain (Duda et al., 2013). Eleven taxonomic quasi‐primes are identified in the NDST1 protein at regions related to calcium and adenosine‐3′‐5′‐diphosphate (A3P) binding. Particularly, the residues His389 and His393 both interact with the calcium ion through coordination covalent bonds, while Phe816 interacts with A3P through π‐stacking interactions (Figure 8c). The sequence coordinates of taxonomic quasi‐primes overlap with the sulfotransferase and the deacetylase active sites, which are known to stabilize the tertiary structure, enabling the correct placement of the N‐acetyl‐heparan sulfate (Mycroft‐West et al., 2024). Five quasi‐primes were identified in the MYH7 protein, interacting with the myosin regulatory light chain 2, ventricular/cardiac muscle isoform (MLC2v) (Figure 8d) through one hydrogen bond at the Trp827 site and electrostatic interactions at the positively charged Lys831. This interaction is found to regulate motor function, stabilize the myosin complex, and enhance calcium sensitivity, ensuring efficient cardiac muscle contraction (Rayment et al., 1993).

This functional importance is not limited only to human proteins. MSA results display that the highly conserved taxonomic quasi‐primes are present within the same coordinates across Chordata orthologs (Figure 8). This finding is a strong indicator of purifying selection, suggesting that these specific peptide sequences are indispensable for the protein's function and have been maintained throughout evolution.

### 3.7 The uniqueness of taxonomic quasi‐primes is highly dependent on exact sequence identity

After having successfully identified taxonomic quasi‐primes across superkingdoms, kingdoms, and phyla, we set out to test if this uniqueness expanded to biochemical properties other than sequence motifs. We focused on the Chordata quasi‐prime seven‐mers with an ε‐score greater than 90.00%. This set of taxonomic quasi‐primes amounted to 2,652 unique peptides. Using the BLOSUM62 substitution matrix, we generated all biologically conservative single amino‐acid variants, where the BLOSUM score was above 0, which were later mapped against all 24,073 reference proteomes.

Of the 37,330 unique conservative variants generated, 67.27% were found in at least one proteome outside of Chordata, 23.72% were mapped to no proteins, and only 9.01% were identified exclusively within the Chordata phylum (Supplementary Figure 7b). In total, these variants appeared in 1,007,246 proteins. 83.41% of these were Chordata proteins, followed by 2.92% in Arthropoda, 2.27% in Streptophyta, and 1.66% in Ascomycota, all eukaryotic phyla (Supplementary Figure 7a). For each original Chordata quasi‐prime, the vast majority of its peptide variants mapped to eukaryotic proteins (Supplementary Figure 7c). We found three taxonomic quasi‐prime seven‐mers, whose variants mapped exclusively to eukaryotic proteins. These peptides were CCEEWVC with 11 variants, CPKRCVC with 8 variants, and CCTPHRT with six variants. The taxonomic quasi‐prime HEQCCWT, with its 11 variants, was identified exclusively in Chordata species, exhibiting an ε‐score of 93.23%. Notably, none of its variants mapped to any proteins across the four superkingdoms. This specific taxonomic quasi‐prime is situated within the human H(+)/Cl(−) exchange transporter 4, spanning sequence coordinates 111–117.

### 3.8 Pathogenic variants are enriched at human taxonomic quasi‐prime loci

**FIGURE 9:**
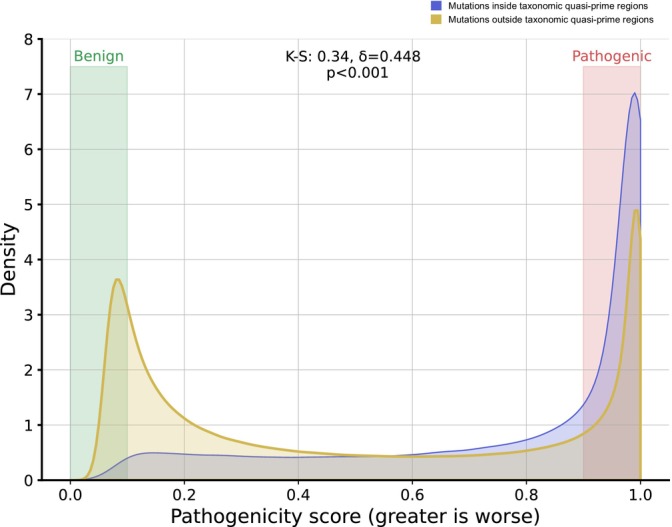
Missense mutations in highly conserved quasi‐prime regions are more likely to be pathogenic. The kernel density estimate plot compares the distribution of AlphaMissense‐predicted pathogenicity scores for missense mutations within human taxonomic quasi‐prime loci (for Chordata quasi‐prime seven‐mers with ε‐scores above 90%) to those outside these loci within the same proteins. Highlighted ranges indicate highly benign mutations (pathogenicity scores <0.1) and highly pathogenic mutations (pathogenicity scores >0.9). Statistical metrics: Kolmogorov–Smirnov (K–S) statistic, Cliff's delta (δ), and *p*‐value (*p*).

Finally, we examined if taxonomic quasi‐prime loci are more likely to harbor pathogenic variants than surrounding sequences. We used human proteome‐wide missense variant effect prediction maps (Cheng et al., 2023), provided by AlphaMissense, and investigated if the subset of missense variants that are pathogenic is more likely to be found at taxonomic quasi‐prime loci. We find that the distribution of variants overlapping taxonomic quasi‐primes is significantly sifted toward pathogenic effects (Kolmogorov–Smirnov test *p*‐value <0.001, Cliff's delta = 0.448) with taxonomic quasi‐prime loci being 2.08‐fold more likely to overlap pathogenic variants than expected (Figure 9). The finding that pathogenic missense variants are significantly enriched within taxonomic quasi‐prime loci underscores their critical importance to protein function. This strong link between lineage‐specific sequences and functional significance makes these regions valuable targets for therapeutic development, including small‐molecule inhibitors or highly specific antibody‐based therapies. From a diagnostic perspective, this implies that mutations within the genomic regions that encode taxonomic quasi‐primes should be prioritized during clinical variant analysis to better assess disease risk and a patient's health status.

## 4 DISCUSSION

The presence of short peptide k‐mers that are highly specific to individual taxonomies has not been studied to date. Here, we provide evidence that based on the tens of thousands of available reference proteomes, such sequences can be systematically discovered across taxonomic levels. We identify peptides that exhibit remarkable taxonomic uniqueness at six‐ and seven‐amino‐acid lengths, offering insights into evolutionary divergence, lineage‐specific adaptations, and proteomic diversity.

We observe large variations in the number and frequency of taxonomic quasi‐primes when comparing different taxonomies. Protista display substantially lower ε_max_ scores when compared to other eukaryotic kingdoms, indicating that their characteristic taxonomic quasi‐prime peptides appear only in a small subset of the corresponding species. This can be attributed to the highly diverse polyphyletic nature of Protista, which encompasses both multicellular and unicellular organisms. Expanding on this, Protista exhibit a wide variety of environments, from marine and terrestrial ecosystems to parasitic niches, increasing the need to adopt different mechanisms for survivability (Burki et al., 2021).

The identification of taxonomic quasi‐primes offers a novel way to study sequence divergence, speciation, and trait development. By identifying k‐mer peptides unique to specific taxa, these sequences can serve as molecular markers of evolutionary processes and lineage‐specific adaptations. Analyzing the proteins harboring taxonomic quasi‐prime peptides may reveal how unique traits emerge, such as specialized metabolic pathways or complex physiological processes. Future research could integrate phylogenetic analyses with taxonomic quasi‐prime peptide distributions to trace evolutionary histories and uncover the genetic basis of speciation events, shedding light on how sequence‐level variations drive biological diversity and the evolution of novel traits.

Tertiary analysis revealed that taxonomic quasi‐primes are not randomly located within the protein structure. Instead, they are preferentially identified in functionally critical regions, where they also facilitate direct roles in the protein's function. They are involved in catalysis and structural stability; they stabilize enzymatic active sites, they mediate essential protein–protein interactions, and finally, they contribute to the stable conformation of large protein complexes.

Although we could not pinpoint any taxonomic quasi‐primes with all variants exclusively within the Chordata phylum, our examination of variant peptides from highly conserved Chordata quasi‐prime seven‐mers revealed that 1,740 out of 2,652 taxonomic quasi‐primes had some corresponding variants exclusively mapped to Chordata and present in our original dataset. To illustrate this, we selected the peptide MWDCMEV (ε‐score = 98.27%), which had 16 variants, 8 of which were specific to the Chordata phylum. This selection was based on MWDCMEV having one of the highest numbers of Chordata‐specific variants and the highest ε‐score.

We propose transitioning from exact taxonomic quasi‐prime sequences to a consensus taxonomic quasi‐prime pattern that encompasses these variants. For instance, the MWDCMEV peptide can be reformatted into the taxonomic quasi‐prime pattern [LM][FW][DEN]CM[EKQ][ILV], where bracketed amino acids represent possible residues at that position. This approach aligns with previous work on pattern‐based categorization, such as'seqlets’ (Smith & Smith, 1990), recurring, conserved amino acid patterns discovered from large, unaligned protein databases using the TEIRESIAS algorithm (Rigoutsos et al., 1999). A similar methodology could advance the concept of taxonomic quasi‐primes toward a pattern‐based direction.

**FIGURE 10:**
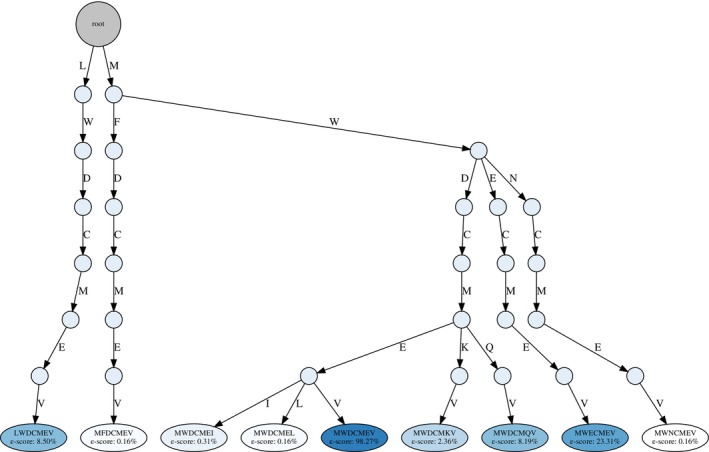
Prefix tree for the Chordata quasi‐prime MWDCMEV. End nodes are colored based on ε‐score, with higher scores having dark blue color.

Furthermore, inspired by the Aho‐Corasick algorithm, these taxonomic quasi‐prime patterns can be represented as prefix trees for efficient taxonomic categorization of newly discovered proteomes. A curated set of highly conserved taxonomic quasi‐primes, each with its variants represented as a prefix tree (Figure 10), can be created for each taxonomic rank. By scanning a novel proteome for these patterns, we can generate a quantitative taxonomic profile for the organism. Counting pattern hits for each major taxonomy (e.g., Chordata, Arthropoda, Bacteria) allows us to calculate a likelihood score, assigning the novel proteome to its most probable lineage. This offers a powerful and efficient method for sequence‐based classification, where matches with higher ε‐scores provide information about the general rank, and associated matches with lower ε‐scores indicate the closest species within that taxonomic rank.

Taxonomic quasi‐prime applications are not limited to just taxonomic classification. Immunological methods based on antibodies can face limitations in sensitivity and specificity, particularly due to cross‐reactivity (Wild, 2013). By prioritizing antigens that harbor taxonomic quasi‐prime peptides, the sensitivity and specificity of designed antibodies can be increased. Given their short length and unique taxonomic specificity, taxonomic quasi‐prime peptides are well suited for integration into mass‐spectrometry workflows (Birhanu, 2023; Dupree et al., 2020). Their inherent specificity can enhance the precision of proteomic analyses, enabling more efficient identification of proteins in complex biological samples (Li et al., 2023). In agriculture, taxonomic quasi‐prime peptides can serve as highly specific biomarkers for pathogen detection. Examination of taxonomic quasi‐prime peptides specific to microbial communities or ecological niches could also provide insights into microbiome dynamics and their role in ecosystem function. In diagnostics, taxonomic quasi‐prime peptides could facilitate the identification of pathogenic organisms in clinical samples, contributing to faster and more precise diagnostics for infectious diseases.

The number of available proteomes represents only a small subset of the species known. Therefore, future work is required to examine how these findings change as more reference proteomes of different organisms become available. The use of TrEMBL proteins limits our findings to some extent, further necessitating the need for future work where highest quality protein annotations will be present. Furthermore, the incorporation of protein isoforms and population variants could influence our conclusions, and further exploration toward these directions is needed, particularly in eukaryotic proteomes, when such data become available across multiple organisms.

## AUTHOR CONTRIBUTIONS

**Eleftherios Bochalis:** Conceptualization; writing – original draft; writing – review and editing; visualization; methodology; software; data curation; formal analysis; validation; investigation. **Michail Patsakis:** Methodology; software; data curation; formal analysis; writing – original draft. **Nikol Chantzi:** Conceptualization; investigation; validation; methodology. **Ioannis Mouratidis:** Writing – review and editing; funding acquisition; supervision; validation; investigation. **Dionysios V. Chartoumpekis:** Writing – review and editing; supervision. **Ilias Georgakopoulos‐Soares:** Supervision; writing – original draft; writing – review and editing; funding acquisition; project administration; validation; conceptualization.

## CONFLICT OF INTEREST STATEMENT

The authors declare no competing interests.

## Supporting information


Supplementary File 1:

Supplementary File 2:

Supplementary File 3:

**Data S1:** Supporting Information

## Data Availability

The data that support the findings of this study are openly available in Taxonomic quasi‐prime raw data at https://zenodo.org/records/14385095. Code used for this study is openly available at https://github.com/Georgakopoulos-Soares-lab/taxonomic_quasi_primes.

## References

[pro70241-bib-0001] Agresti A . On logit confidence intervals for the odds ratio with small samples. Biometrics. 1999;55:597–602. 11318220 10.1111/j.0006-341x.1999.00597.x

[pro70241-bib-0002] Al‐Amrani S , Al‐Jabri Z , Al‐Zaabi A , Alshekaili J , Al‐Khabori M . Proteomics: concepts and applications in human medicine. World J Biol Chem. 2021;12:57–69. 34630910 10.4331/wjbc.v12.i5.57 PMC8473418

[pro70241-bib-0003] Ashburner M , Ball CA , Blake JA , Botstein D , Butler H , Cherry JM , et al. Gene ontology: tool for the unification of biology. Nat Genet. 2000;25:25–29. 10802651 10.1038/75556 PMC3037419

[pro70241-bib-0004] Balbi T , Ciacci C , Canesi L . Estrogenic compounds as exogenous modulators of physiological functions in molluscs: signaling pathways and biological responses. Comp Biochem Physiol C Toxicol Pharmacol. 2019;222:135–144. 31055067 10.1016/j.cbpc.2019.05.004

[pro70241-bib-0005] Berman HM , Westbrook J , Feng Z , Gilliland G , Bhat TN , Weissig H , et al. The Protein Data Bank. Nucleic Acids Res. 2000;28:235–242. 10592235 10.1093/nar/28.1.235 PMC102472

[pro70241-bib-0006] Bettembourg C , Diot C , Dameron O . Optimal threshold determination for interpreting semantic similarity and particularity: application to the comparison of gene sets and metabolic pathways using GO and ChEBI. PLoS One. 2015;10:e0133579. 26230274 10.1371/journal.pone.0133579 PMC4521860

[pro70241-bib-0007] Birhanu AG . Mass spectrometry‐based proteomics as an emerging tool in clinical laboratories. Clin Proteomics. 2023;20:1–20. 37633929 10.1186/s12014-023-09424-x PMC10464495

[pro70241-bib-0008] Burki F , Sandin MM , Jamy M . Diversity and ecology of protists revealed by metabarcoding. Curr Biol. 2021;31:R1267–R1280. 34637739 10.1016/j.cub.2021.07.066

[pro70241-bib-0009] Chantzi N , Mareboina M , Konnaris MA , Montgomery A , Patsakis M , Mouratidis I , et al. The determinants of the rarity of nucleic and peptide short sequences in nature. NAR Genom Bioinform. 2024;6:lqae029. 38584871 10.1093/nargab/lqae029 PMC10993293

[pro70241-bib-0010] Chapman JR . Protein and peptide analysis by mass spectrometry. New Jersey, United States: Humana Press; 2013.

[pro70241-bib-0011] Chen C , Li Z , Huang H , Suzek BE , Wu CH , UniProt Consortium . A fast peptide match service for UniProt knowledgebase. Bioinformatics. 2013;29:2808–2809. 23958731 10.1093/bioinformatics/btt484 PMC3799477

[pro70241-bib-0012] Cheng J , Novati G , Pan J , Bycroft C , Žemgulytė A , Applebaum T , et al. Accurate proteome‐wide missense variant effect prediction with AlphaMissense. Science. 2023; 381(6664):1284–1295. 10.1126/science.adg7492 37733863

[pro70241-bib-0013] Cheng N , Brantley DM , Chen J . The ephrins and Eph receptors in angiogenesis. Cytokine Growth Factor Rev. 2002;13:75–85. 11750881 10.1016/s1359-6101(01)00031-4

[pro70241-bib-0014] Dey D , Dhar D , Das S , Maulik A , Basu S . Structural and functional implications of leucine‐rich repeats in toll‐like receptor1 subfamily. J Biosci. 2022; 47:59: Available from: https://www.ncbi.nlm.nih.gov/pubmed/36222137 36222137

[pro70241-bib-0015] Duda DM , Olszewski JL , Schuermann JP , Kurinov I , Miller DJ , Nourse A , et al. Structure of HHARI, a RING‐IBR‐RING ubiquitin ligase: autoinhibition of an Ariadne‐family E3 and insights into ligation mechanism. Structure. 2013;21:1030–1041. 23707686 10.1016/j.str.2013.04.019 PMC3747818

[pro70241-bib-0016] Dupree EJ , Jayathirtha M , Yorkey H , Mihasan M , Petre BA , Darie CC . A critical review of bottom‐up proteomics: the good, the bad, and the future of this field. Proteomes. 2020;8:14. 10.3390/proteomes8030014 32640657 PMC7564415

[pro70241-bib-0017] Frey PA , Hegeman AD , Ruzicka FJ . The radical SAM superfamily. Crit Rev Biochem Mol Biol. 2008;43:63–88. 18307109 10.1080/10409230701829169

[pro70241-bib-0018] Frishman D , Argos P . Knowledge‐based protein secondary structure assignment. Proteins. 1995;23:566–579. 8749853 10.1002/prot.340230412

[pro70241-bib-0019] Gene Ontology Consortium , Aleksander SA , Balhoff J , Carbon S , Cherry JM , Drabkin HJ , et al. The gene ontology knowledgebase in 2023. Genetics. 2023;224(1):iyad031. 10.1093/genetics/iyad031 36866529 PMC10158837

[pro70241-bib-0020] Georgakopoulos‐Soares I , Yizhar‐Barnea O , Mouratidis I , Hemberg M , Ahituv N . Absent from DNA and protein: genomic characterization of nullomers and nullpeptides across functional categories and evolution. Genome Biol. 2021;22:245. 34433494 10.1186/s13059-021-02459-z PMC8386077

[pro70241-bib-0021] Hajisharifi Z , Piryaiee M , Mohammad Beigi M , Behbahani M , Mohabatkar H . Predicting anticancer peptides with Chou's pseudo amino acid composition and investigating their mutagenicity via Ames test. J Theor Biol. 2014;341:34–40. 24035842 10.1016/j.jtbi.2013.08.037

[pro70241-bib-0022] Henikoff S , Henikoff JG . Amino acid substitution matrices from protein blocks. Proc Natl Acad Sci USA. 1992;89:10915–10919. 1438297 10.1073/pnas.89.22.10915 PMC50453

[pro70241-bib-0023] Huerta‐Cepas J , Szklarczyk D , Heller D , Hernández‐Plaza A , Forslund SK , Cook H , et al. eggNOG 5.0: a hierarchical, functionally and phylogenetically annotated orthology resource based on 5090 organisms and 2502 viruses. Nucleic Acids Res. 2019;47:D309–D314. 30418610 10.1093/nar/gky1085 PMC6324079

[pro70241-bib-0024] Jones MDM , Forn I , Gadelha C , Egan MJ , Bass D , Massana R , et al. Discovery of novel intermediate forms redefines the fungal tree of life. Nature. 2011;474:200–203. 21562490 10.1038/nature09984

[pro70241-bib-0025] Jumper J , Evans R , Pritzel A , Green T , Figurnov M , Ronneberger O , et al. Highly accurate protein structure prediction with AlphaFold. Nature. 2021;596:583–589. 34265844 10.1038/s41586-021-03819-2 PMC8371605

[pro70241-bib-0026] Kamke J , Rinke C , Schwientek P , Mavromatis K , Ivanova N , Sczyrba A , et al. The candidate phylum Poribacteria by single‐cell genomics: new insights into phylogeny, cell‐compartmentation, eukaryote‐like repeat proteins, and other genomic features. PLoS One. 2014;9:e87353. 24498082 10.1371/journal.pone.0087353 PMC3909097

[pro70241-bib-0027] Kao T‐J , Kania A . Ephrin‐mediated cis‐attenuation of Eph receptor signaling is essential for spinal motor axon guidance. Neuron. 2011;71:76–91. 21745639 10.1016/j.neuron.2011.05.031

[pro70241-bib-0028] Katoh K , Standley DM . MAFFT multiple sequence alignment software version 7: improvements in performance and usability. Mol Biol Evol. 2013;30:772–780. 23329690 10.1093/molbev/mst010 PMC3603318

[pro70241-bib-0029] Klopfenstein DV , Zhang L , Pedersen BS , Ramírez F , Warwick Vesztrocy A , Naldi A , et al. GOATOOLS: a python library for gene ontology analyses. Sci Rep. 2018;8:10872. 30022098 10.1038/s41598-018-28948-z PMC6052049

[pro70241-bib-0030] Krasowski MD , Yasuda K , Hagey LR , Schuetz EG . Evolutionary selection across the nuclear hormone receptor superfamily with a focus on the NR1I subfamily (vitamin D, pregnane X, and constitutive androstane receptors). Nucl Recept. 2005;3:2. 16197547 10.1186/1478-1336-3-2 PMC1262763

[pro70241-bib-0031] Kullander K , Mather NK , Diella F , Dottori M , Boyd AW , Klein R . Kinase‐dependent and kinase‐independent functions of EphA4 receptors in major axon tract formation in vivo. Neuron. 2001;29:73–84. 11182082 10.1016/s0896-6273(01)00181-7

[pro70241-bib-0032] Lacerda CMR , Reardon KF . Environmental proteomics: applications of proteome profiling in environmental microbiology and biotechnology. Brief Funct Genomic Proteomic. 2009;8:75–87. 19279070 10.1093/bfgp/elp005

[pro70241-bib-0033] Li L , Wang T , Ning Z , Zhang X , Butcher J , Serrana JM , et al. Revealing proteome‐level functional redundancy in the human gut microbiome using ultra‐deep metaproteomics. Nat Commun. 2023;14:3428. 37301875 10.1038/s41467-023-39149-2 PMC10257714

[pro70241-bib-0034] Markson JS , O'Shea EK . The molecular clockwork of a protein‐based circadian oscillator. FEBS Lett. 2009;583:3938–3947. 19913541 10.1016/j.febslet.2009.11.021 PMC2810098

[pro70241-bib-0035] Matsushima N , Miyashita H , Kretsinger RH . Sequence features, structure, ligand interaction, and diseases in small leucine rich repeat proteoglycans. J Cell Commun Signal. 2021;15:519–531. 33860400 10.1007/s12079-021-00616-4 PMC8642563

[pro70241-bib-0036] McInnes L , Healy J , Melville J . UMAP: uniform manifold approximation and projection for dimension reduction. 2018 Available from: http://arxiv.org/abs/1802.03426

[pro70241-bib-0037] McMahon HT , Boucrot E . Molecular mechanism and physiological functions of clathrin‐mediated endocytosis. Nat Rev Mol Cell Biol. 2011;12:517–533. 21779028 10.1038/nrm3151

[pro70241-bib-0038] Meng EC , Goddard TD , Pettersen EF , Couch GS , Pearson ZJ , Morris JH , et al. UCSF ChimeraX: tools for structure building and analysis. Protein Sci. 2023;32:e4792. 37774136 10.1002/pro.4792 PMC10588335

[pro70241-bib-0039] Moeckel C , Mareboina M , Konnaris MA , Chan CSY , Mouratidis I , Montgomery A , et al. A survey of k‐mer methods and applications in bioinformatics. Comput Struct Biotechnol J. 2024;23:2289–2303. 38840832 10.1016/j.csbj.2024.05.025 PMC11152613

[pro70241-bib-0040] Mouratidis I , Baltoumas FA , Chantzi N , Patsakis M , Chan CSY , Montgomery A , et al. kmerDB: a database encompassing the set of genomic and proteomic sequence information for each species. Comput Struct Biotechnol J. 2024;23:1919–1928. 38711760 10.1016/j.csbj.2024.04.050 PMC11070822

[pro70241-bib-0041] Mouratidis I , Chan CSY , Chantzi N , Tsiatsianis GC , Hemberg M , Ahituv N , et al. Quasi‐prime peptides: identification of the shortest peptide sequences unique to a species. NAR Genom Bioinform. 2023;5:lqad039. 37101657 10.1093/nargab/lqad039 PMC10124967

[pro70241-bib-0042] Mycroft‐West CJ , Abdelkarim S , Duyvesteyn HME , Gandhi NS , Skidmore MA , Owens RJ , et al. Structural and mechanistic characterization of bifunctional heparan sulfate N‐deacetylase‐N‐sulfotransferase 1. Nat Commun. 2024;15:1–17. 38351061 10.1038/s41467-024-45419-4 PMC10864358

[pro70241-bib-0043] Ohama T . The multiple functions of protein phosphatase 6. Biochim Biophys Acta Mol Cell Res. 2019;1866:74–82. 30036567 10.1016/j.bbamcr.2018.07.015

[pro70241-bib-0044] Pao SS , Paulsen IT , Saier MH Jr . Major facilitator superfamily. Microbiol Mol Biol Rev. 1998;62:1–34. 9529885 10.1128/mmbr.62.1.1-34.1998 PMC98904

[pro70241-bib-0045] Paysan‐Lafosse T , Blum M , Chuguransky S , Grego T , Pinto BL , Salazar GA , et al. InterPro in 2022. Nucleic Acids Res https://pubmed.ncbi.nlm.nih.gov/36350672/. 2023;51:D418–D427. 36350672 10.1093/nar/gkac993 PMC9825450

[pro70241-bib-0046] Rayment I , Rypniewski WR , Schmidt‐Bäse K , Smith R , Tomchick DR , Benning MM , et al. Three‐dimensional structure of myosin subfragment‐1: a molecular motor. Science. 1993;261:50–58. 8316857 10.1126/science.8316857

[pro70241-bib-0047] Rigoutsos I , Floratos A , Ouzounis C , Gao Y , Parida L . Dictionary building via unsupervised hierarchical motif discovery in the sequence space of natural proteins. Proteins. 1999;37:264–277. Available from: https://pubmed.ncbi.nlm.nih.gov/10584071/ 10584071 10.1002/(sici)1097-0134(19991101)37:2<264::aid-prot11>3.0.co;2-c

[pro70241-bib-0048] Sarrazin S , Lamanna WC , Esko JD . Heparan sulfate proteoglycans. Cold Spring Harb Perspect Biol. 2011;3:a004952. 10.1101/cshperspect.a004952 21690215 PMC3119907

[pro70241-bib-0049] Smith RF , Smith TF . Automatic generation of primary sequence patterns from sets of related protein sequences. Proc Natl Acad Sci USA. 1990;87:118–122. 2296575 10.1073/pnas.87.1.118 PMC53211

[pro70241-bib-0050] Steenwyk JL , Buida TJ 3rd , Li Y , Shen X‐X , Rokas A . ClipKIT: a multiple sequence alignment trimming software for accurate phylogenomic inference. PLoS Biol. 2020;18:e3001007. 33264284 10.1371/journal.pbio.3001007 PMC7735675

[pro70241-bib-0051] UniProt Consortium . UniProt: the universal protein knowledgebase in 2023. Nucleic Acids Res. 2023;51:D523–D531. 36408920 10.1093/nar/gkac1052 PMC9825514

[pro70241-bib-0052] ValizadehAslani T , Zhao Z , Sokhansanj BA , Rosen GL . Amino acid ‐mer feature extraction for quantitative antimicrobial resistance (AMR) prediction by machine learning and model interpretation for biological insights. Biology. 2020;9:365. 10.3390/biology9110365 33126516 PMC7694136

[pro70241-bib-0053] Varadi M , Bertoni D , Magana P , Paramval U , Pidruchna I , Radhakrishnan M , et al. AlphaFold protein structure database in 2024: providing structure coverage for over 214 million protein sequences. Nucleic Acids Res. 2024;52:D368–D375. 37933859 10.1093/nar/gkad1011 PMC10767828

[pro70241-bib-0054] Wang JZ , Du Z , Payattakool R , Yu PS , Chen C‐F . A new method to measure the semantic similarity of GO terms. Bioinformatics. 2007;23:1274–1281. 17344234 10.1093/bioinformatics/btm087

[pro70241-bib-0055] Waterhouse AM , Procter JB , Martin DMA , Clamp M , Barton GJ . Jalview version 2—a multiple sequence alignment editor and analysis workbench. Bioinformatics. 2009;25:1189–1191. 19151095 10.1093/bioinformatics/btp033 PMC2672624

[pro70241-bib-0056] Wen J , Chan RHF , Yau S‐C , He RL , Yau SST . K‐mer natural vector and its application to the phylogenetic analysis of genetic sequences. Gene. 2014;546:25–34. 24858075 10.1016/j.gene.2014.05.043 PMC4096558

[pro70241-bib-0057] Wild D . The immunoassay handbook: theory and applications of ligand binding, ELISA and Related techniques. Oxford, United Kingdom: Newnes; 2013.

[pro70241-bib-0058] Wu C , Gao R , Zhang Y , De Marinis Y . PTPD: predicting therapeutic peptides by deep learning and word2vec. BMC Bioinform. 2019;20:456. 10.1186/s12859-019-3006-z PMC6728961 31492094

[pro70241-bib-0059] Yang Q , Zhao J , Chen D , Wang Y . E3 ubiquitin ligases: styles, structures and functions. Mol Biomed. 2021;2:23. 35006464 10.1186/s43556-021-00043-2 PMC8607428

