# Supplementary material for: Taxonomic quasi‐primes: peptides charting lineage‐specific adaptations and disease‐relevant loci
Source: Protein Sci. 2025 Aug 25;34(9):e70241. doi: 10.1002/pro.70241 (PMC12375989; doi:10.1002/pro.70241)
Supplement: Supplementary file 4 — Data S1: Supporting Information [file PRO-34-e70241-s002.docx]

**Supplementary Material**

**
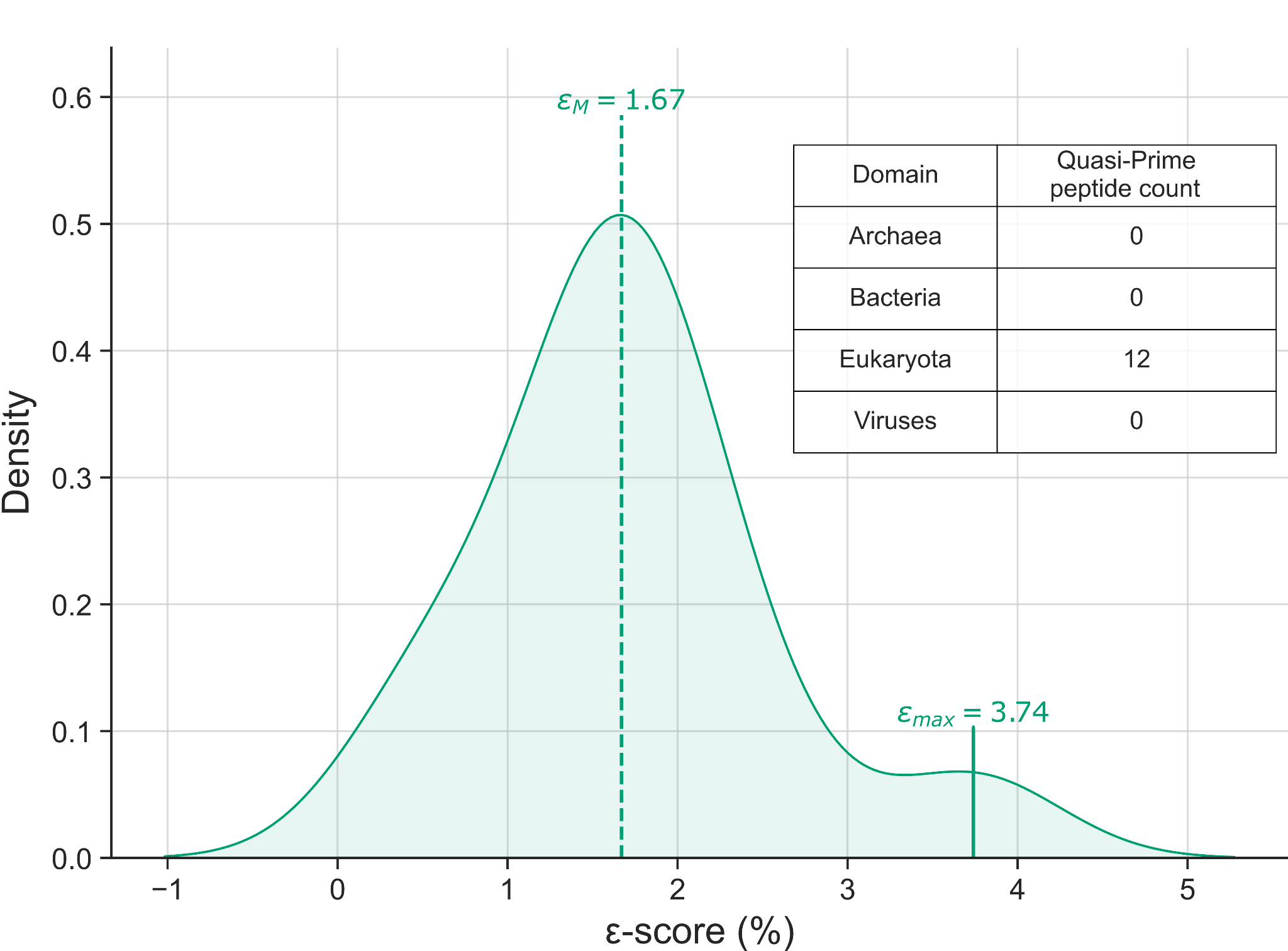
**

**Supplementary Figure 1: Kernel density estimate plot illustrating the ε-score distribution of superkingdom quasi-prime five-mers.** The smoothing of the curve extending into negative values and exceeding ε_max_ is likely due to the limited sample size of peptides, which affects the Gaussian kernel's estimation fidelity.


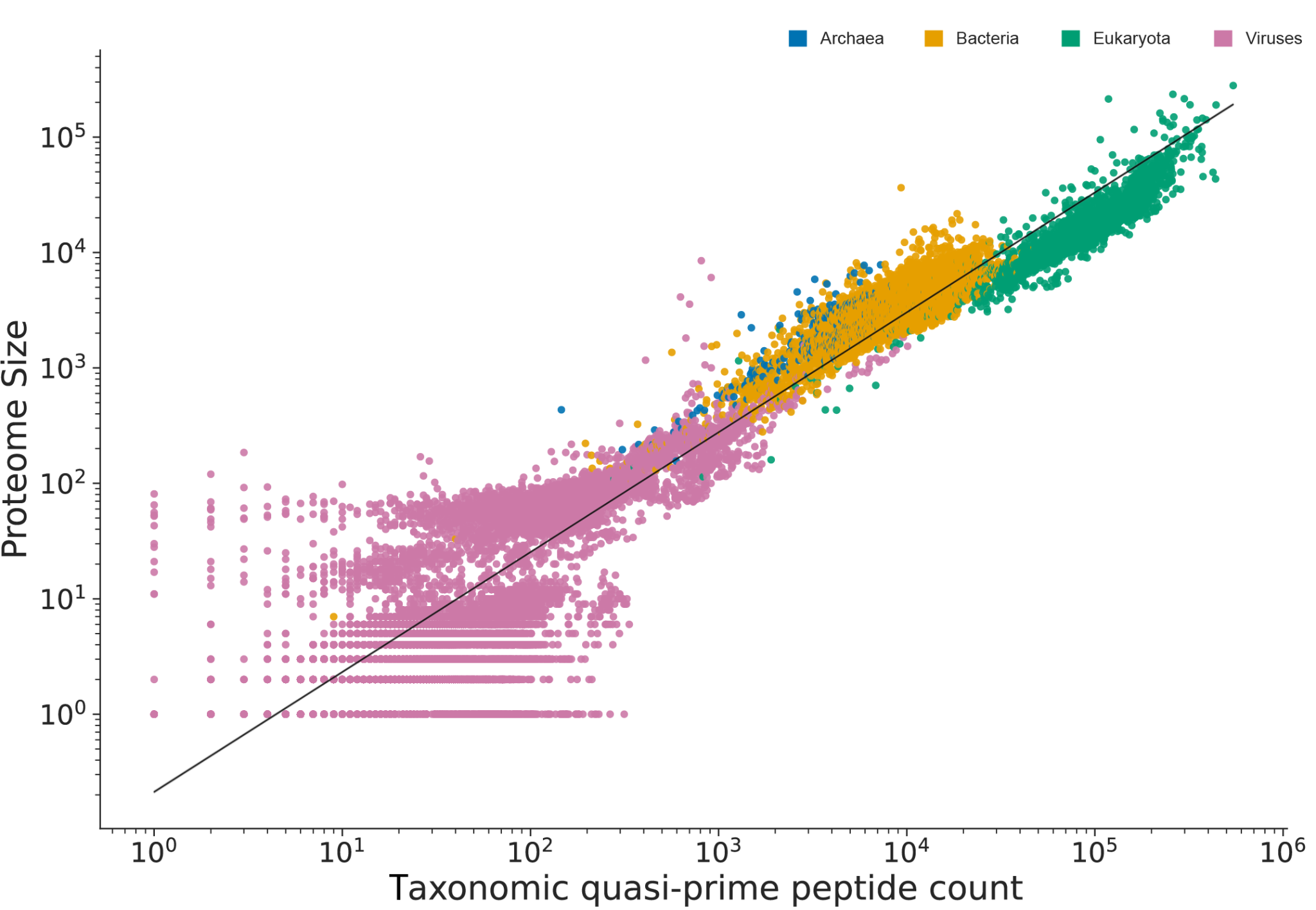


**Supplementary Figure 2: Scatterplot representing the number of unique taxonomic quasi-prime peptides (six-mers and seven-mers) observed in each reference proteome as a function of their proteome size.**

**
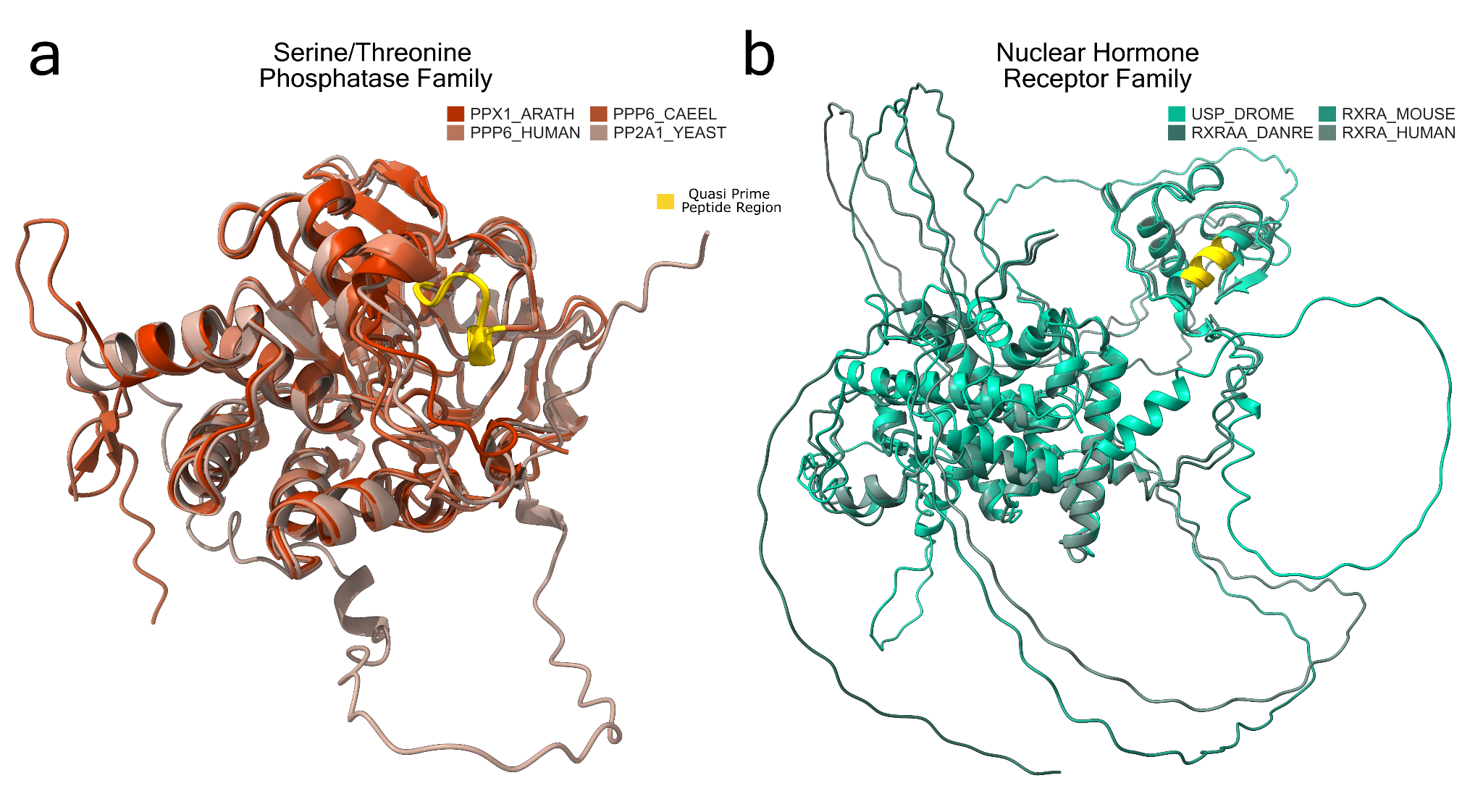
**

**Supplementary Figure 3: Tertiary depiction of taxonomic quasi-primes with the highest ε-score across superkingdom and kingdom.**

**a** Superposition of proteins representing the Serine/Threonine Phosphatase family at the eukaryotic superkingdom. Results are shown for *Arabidopsis thaliana, Caenorhabditis elegans, Homo sapiens*, and *Saccharomyces cerevisiae* orthologs. **b** Superposition of proteins representing the Nuclear Hormone Receptor family at the metazoan kingdom. Results are shown for *Drosophila melanogaster*, *Mus musculus*, *Danio rerio*, and *Homo sapiens* orthologs. Taxonomic quasi-prime peptides are marked in yellow.


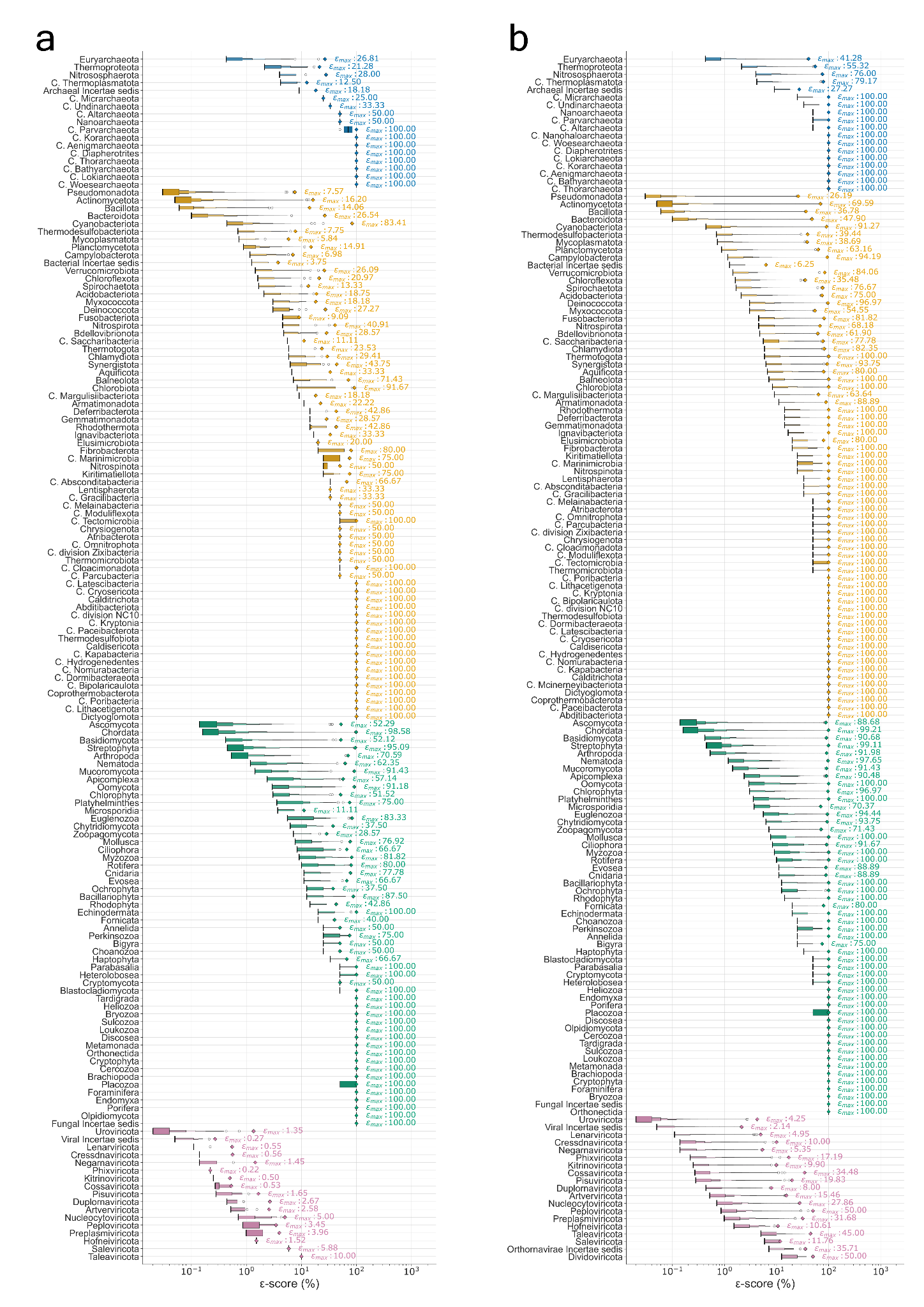


**Supplementary Figure 4: ε-score distribution of taxonomic quasi-prime peptides across all phyla at the superkingdom level.**


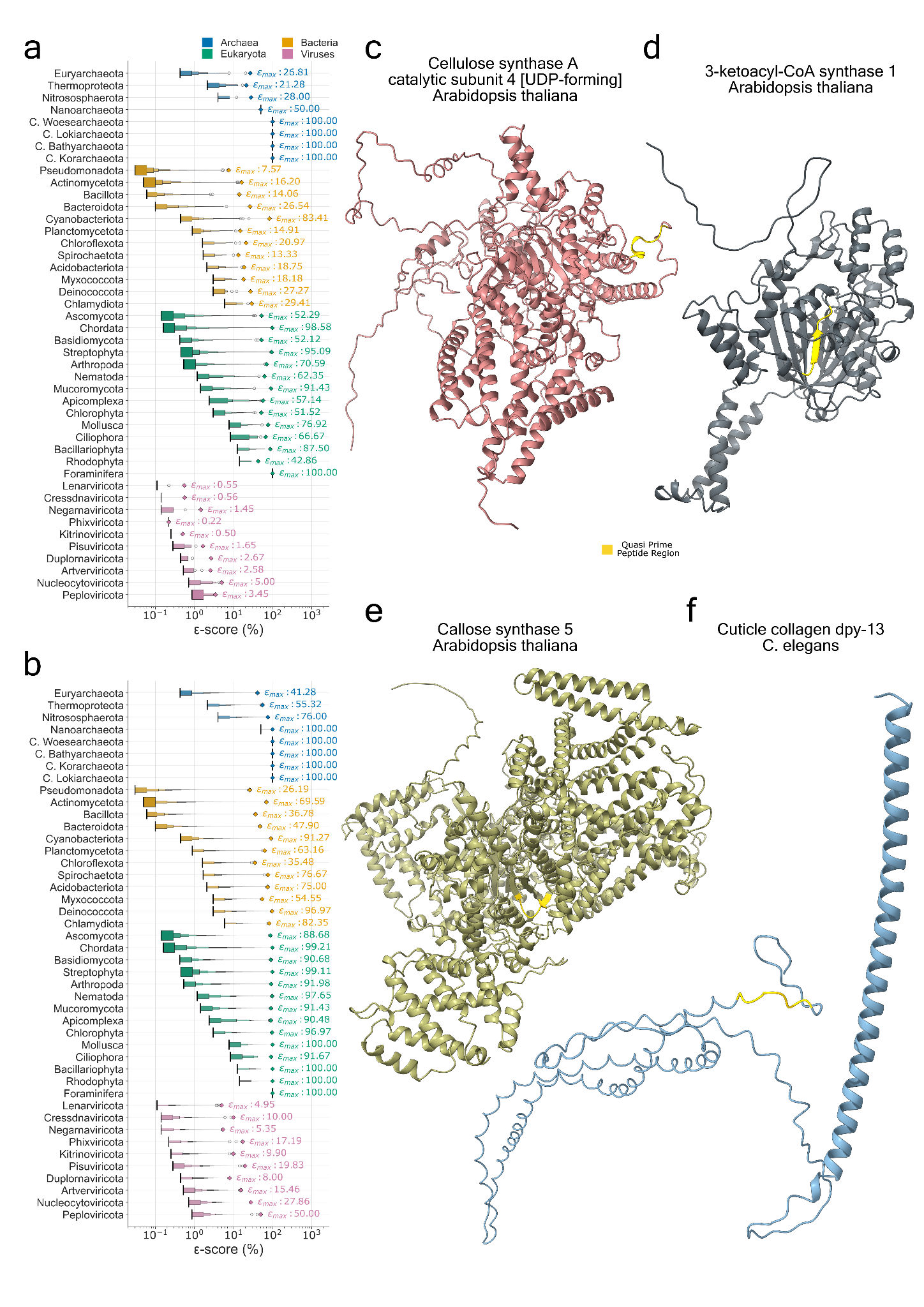


**Supplementary Figure 5: ε-score distribution of quasi-prime peptides across representative phyla at the superkingdom level.** Letter-value plots illustrate the ε-score distributions for representative phyla at the superkingdom level. The ε_M_ value is depicted as a solid black line and the ε_max_ value as a rhombus. Distributions are shown separately for taxonomic quasi-prime peptides of different lengths: **a** Taxonomic quasi-prime six-mers **b** Taxonomic quasi-prime seven-mers. The color represents the corresponding superkingdom. **c-f** Protein structure of: **c** the Cellulose Synthase A Catalytic Subunit 4, **d** 3-Ketoacyl-CoA Synthase 1, **e** Callose synthase 5, in *Arabidopsis thaliana* and cuticle collagen DPY-13 protein of *C. elegans.* Taxonomic quasi-prime peptides are marked in yellow.

**
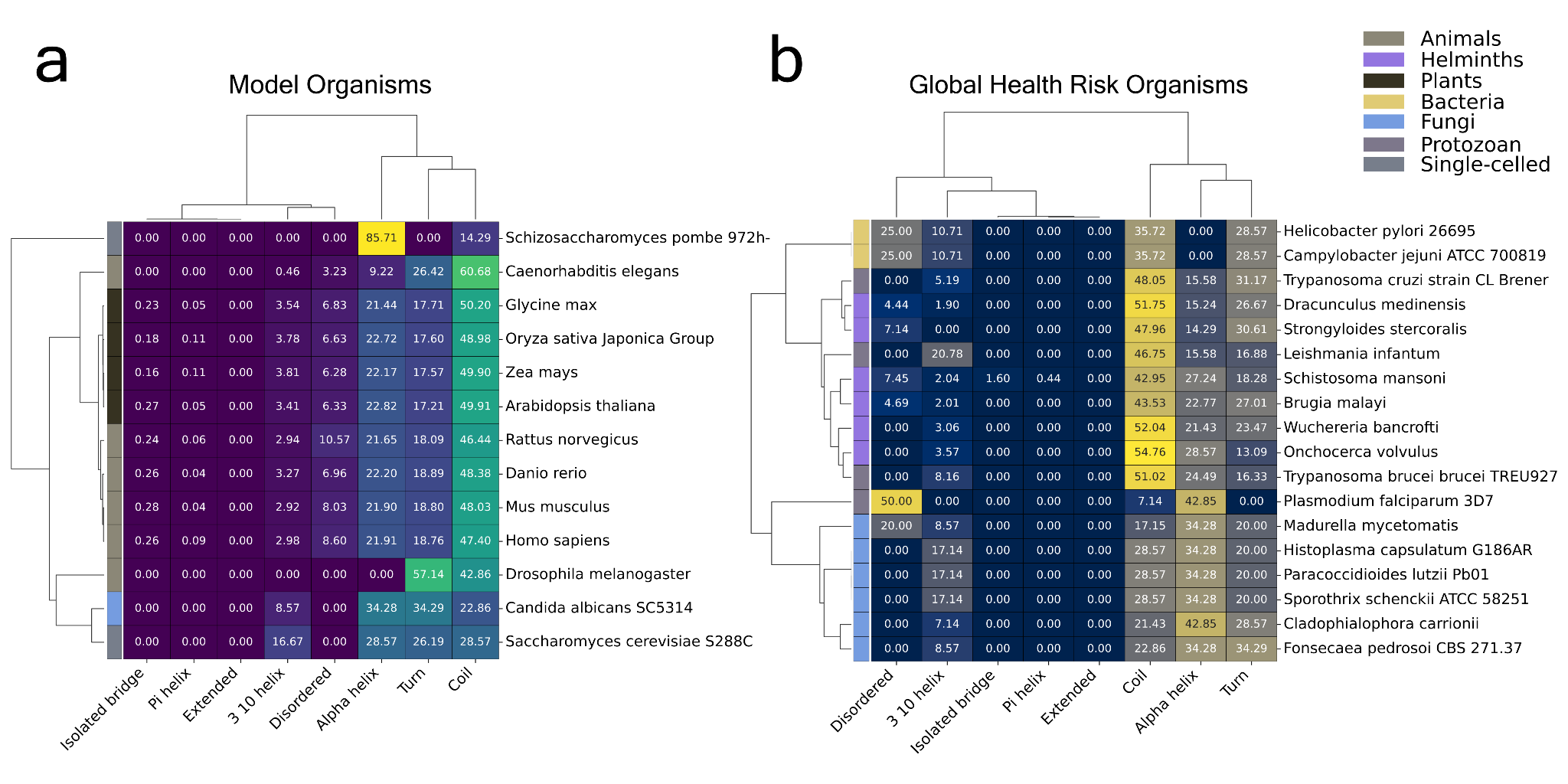
**

**Supplementary Figure 6: Secondary structure profiling of taxonomic quasi-primes in model and global health risk organisms. a** Clustered heatmap illustrating the secondary structure composition of taxonomic quasi-primes with ε-score exceeding 90.00% across selected model organisms. **b** Clustered heatmap of the secondary structure composition of taxonomic quasi-primes with ε-scores over 90.00% in selected global health risk organisms, encompassing Bacteria, Fungi, Helminths, Protozoans, and other single-celled pathogens. Rows (organisms) and columns (secondary structure types) have been hierarchically clustered based on Euclidean distance, employing Ward’s method to reveal patterns and structural similarities among species. Each heatmap cell displays the exact percentage of a specific secondary structure type within each organism.


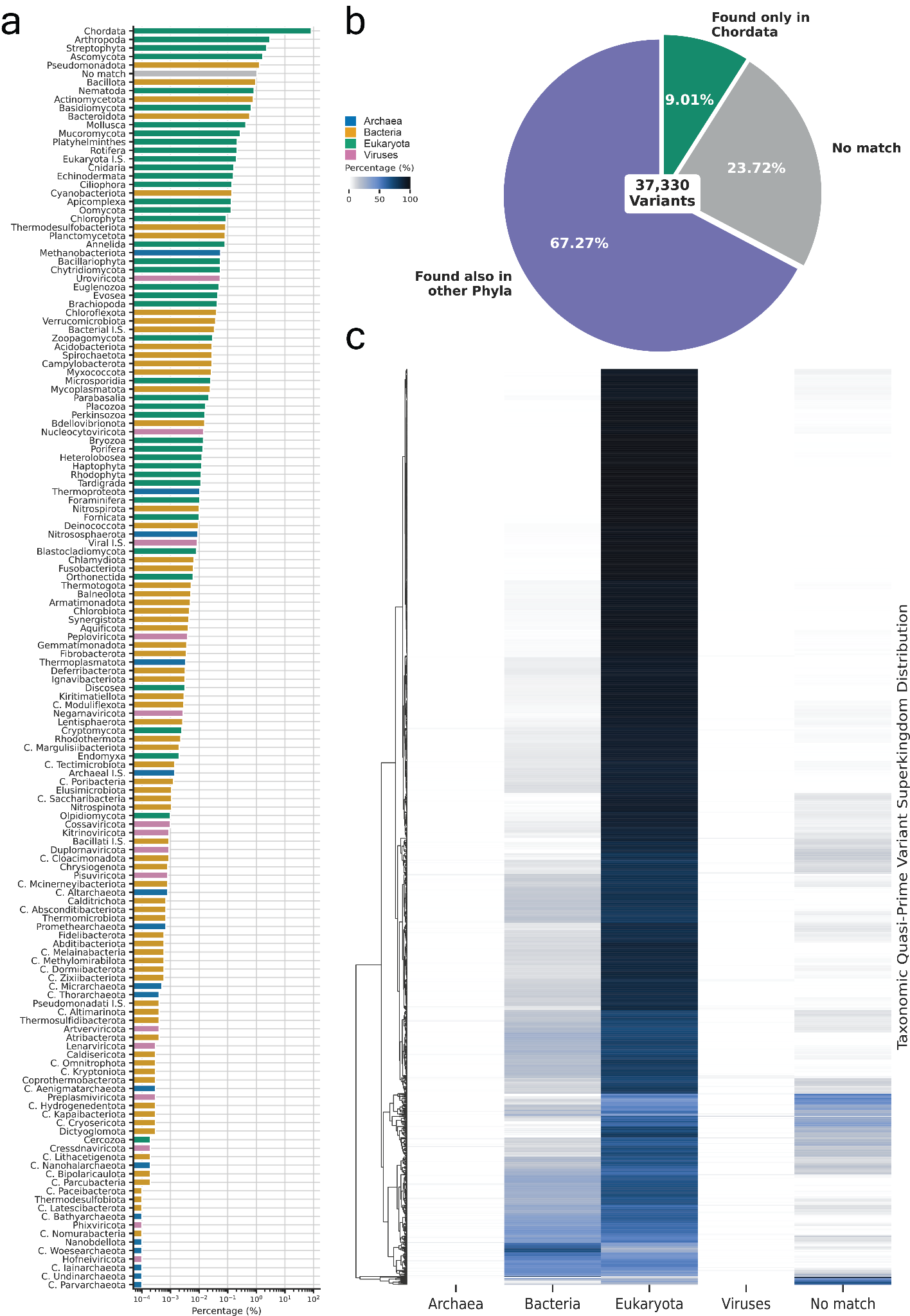


**Supplementary Figure 7: Substitution matrix analysis of highly conserved Chordata quasi-prime variants. a.** Bar plot showing the distribution of identified proteins containing variants across different phyla. The x-axis depicting the percentages is in logarithmic scale. **b.** Pie chart illustrating the percentage of single amino-acid taxonomic quasi-prime variants. These are categorized by whether they remain within the Chordata phylum, occur outside it (if at least one protein matches a different phylum), or have no match. **c.** Clustered heatmap displaying the superkingdom distribution for each original Chordata quasi-prime's variants. Rows correspond to individual taxonomic quasi-primes, and columns represent superkingdoms and values represent the percentage of the variant’s mapped proteins that fall within each superkingdom.

**Supplementary Table 1: List of Eukaryota quasi-prime five-mer sequences identified at the superkingdom level, along with their corresponding ε-scores.**

| **Peptide** | **ε-score (%)** |
| --- | --- |
| HMCWY | 0.52 |
| WCMMW | 0.56 |
| WCHCM | 1.29 |
| WCCMW | 1.41 |
| HYWCC | 1.53 |
| HCHWW | 1.65 |
| CCHWW | 1.69 |
| CHCWM | 1.77 |
| KWHMC | 1.85 |
| CHCMW | 2.05 |
| QYCWC | 2.41 |
| WCMCE | 3.74 |

**Supplementary Table 2: Taxonomic quasi-prime peptides (six-mers and seven-mers) exhibiting the highest ε-score across eukaryotic kingdoms.**

| **Eukaryotic quasi-prime six-mers** | | | **Eukaryotic quasi-prime seven-mers** | | |
| --- | --- | --- | --- | --- | --- |
| Kingdom | Peptide | ε_max_ (%) | Kingdom | Peptide | ε_max_ (%) |
| Fungi | FPKCYW | 34.58 | Fungi | DANQDNY | 92.50 |
| Metazoa | KWMMYW | 87.18 | Metazoa | CKGFFKR | 98.78 |
| Viridiplantae | YPCFMW | 82.88 | Viridiplantae | KSCRLRW | 98.05 |
| Protista | WHDCHC WWEFYH | 17.03 | Protista | REENKWC | 37.91 |

**Supplementary Table 3: Taxonomic quasi-prime seven-mers identified in selected Chordata proteins**

| **Protein** | **Peptide** | **Region in protein** | **ε-score (%)** |
| --- | --- | --- | --- |
| **Human Sodium channel 8A** | ELPRWHM | 913 - 919 | 93.86 |
|  | EWIETMW | 936 - 942 | 98.9 |
|  | WIETMWD | 937 - 943 | 98.74 |
|  | IETMWDC | 938 - 944 | 98.74 |
|  | ETMWDCM | 939 - 945 | 99.21 |
|  | TMWDCME | 940 - 946 | 98.27 |
|  | MWDCMEV | 941 - 947 | 98.27 |
|  | WDCMEVA | 942 - 948 | 98.27 |
|  | IVEHNWF | 1194 - 1200 | 91.02 |
|  | CLNMVTM | 1533 - 1539 | 98.58 |
|  | NMVTMMV | 1535 - 1541 | 98.43 |
|  | YFTIGWN | 1580 - 1586 | 95.12 |
| **Human E3 ubiquitin-protein ligase ARIH2** | CFKCRQM | 257 - 263 | 91.50 |
|  | FKCRQMY | 258 - 264 | 91.97 |
|  | KCRQMYH | 259 - 265 | 91.97 |
| **Human bifunctional heparan sulfate N-deacetylase/N-sulfotransferase 1** | KEFWWFP | 382 - 388 | 90.08 |
|  | WFPHMWS | 386 - 392 | 98.58 |
|  | FPHMWSH | 387 - 393 | 98.58 |
|  | PHMWSHM | 388 - 394 | 98.58 |
|  | HMWSHMQ | 389 - 395 | 98.58 |
|  | MWSHMQP | 390 - 396 | 98.27 |
|  | WQDPCED | 582 - 588 | 90.71 |
|  | CEDKRHK | 586 - 592 | 93.39 |
|  | YLFLGMH | 621 - 627 | 91.50 |
|  | KGFWCQL | 814 - 820 | 96.22 |
|  | FWCQLLE | 816 - 822 | 90.87 |
| **Human Myosin 7** | VKNWPWM | 824 - 830 | 94.17 |
|  | KNWPWMK | 825 - 831 | 93.86 |
|  | NWPWMKL | 826 - 832 | 93.70 |
|  | WPWMKLY | 827 - 833 | 90.71 |
|  | WMKLYFK | 829 - 835 | 90.71 |

**Supplementary information: Random effects meta-analysis for enrichment result combination across species belonging to the same phylum**

A meta-analysis technique employing a random effects model was performed to identify gene ontology terms and functional entries (domains or families) that are present across multiple species of the same phylum and evaluate the combined enrichment of each of them. Common items were filtered based on the criteria described earlier, and the natural logarithm of the OR value (LOR) was calculated. An original fixed-effect weight was calculated for each study (species), so that studies with more precise estimates (smaller standard error) are given a larger weight in the analysis, as follows:

| [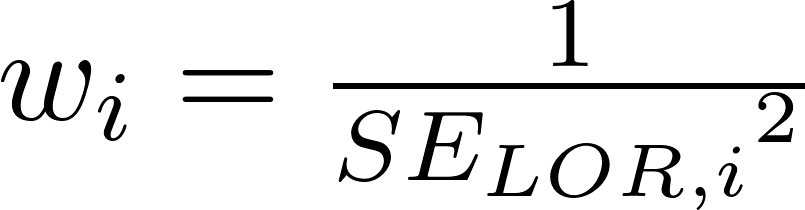](https://www.codecogs.com/eqnedit.php?latex=w_i%20%3D%20%5Cfrac%7B1%7D%7B%7BSE_%7BLOR%2Ci%7D%7D%5E%7B2%7D%7D#0) | (1) |
| --- | --- |

where, [
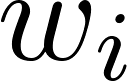
](https://www.codecogs.com/eqnedit.php?latex=w_i#0) represents the weight for the i_th_ study, [
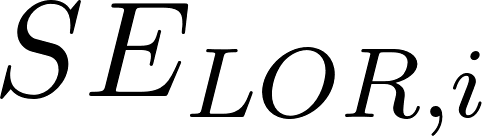
](https://www.codecogs.com/eqnedit.php?latex=SE_%7BLOR%2Ci%7D#0) is the standard error of the LOR value for the ith study and [
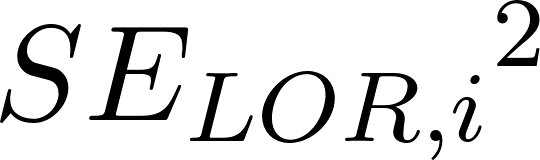
](https://www.codecogs.com/eqnedit.php?latex=%7BSE_%7BLOR%2Ci%7D%7D%5E%7B2%7D#0) is the variance of the LOR estimate for the i_th_ study. The initial combined enrichment was obtained through the computation of the fixed effects weighted mean, which will be later used to assess the heterogeneity between-studies. The calculation of the fixed effects weighted mean goes as follows:

| [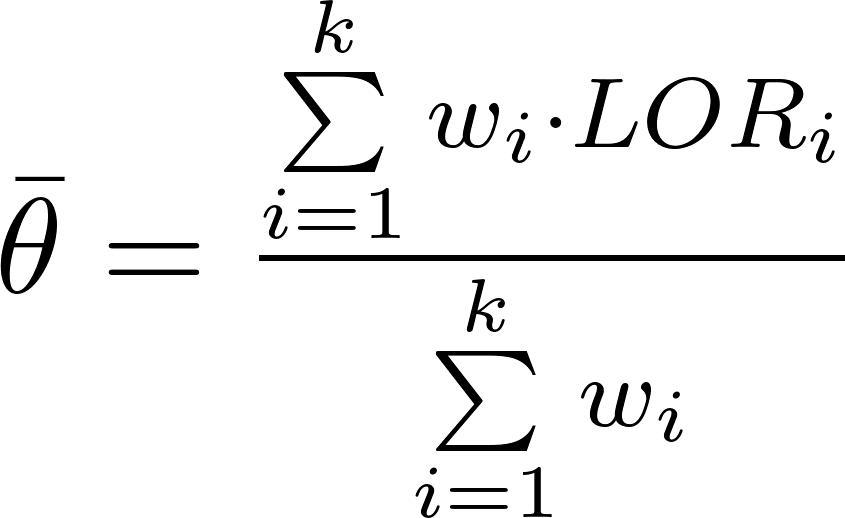](https://www.codecogs.com/eqnedit.php?latex=%5Cbar%7B%5Ctheta%7D%20%3D%20%5Cfrac%7B%5Csum%5Climits_%7Bi%3D1%7D%5E%7Bk%7D%20w_i%5Ccdot%20LOR_i%7D%7B%5Csum%5Climits_%7Bi%3D1%7D%5E%7Bk%7D%20w_i%7D#0) | (2) |
| --- | --- |

where [
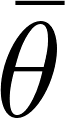
](https://www.codecogs.com/eqnedit.php?latex=%5Cbar%7B%5Ctheta%7D#0) represents the weighted mean LOR using the fixed-effects model and [
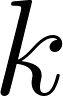
](https://www.codecogs.com/eqnedit.php?latex=k#0) represents the total number of studies. The use of Cochran’s Q statistic was implemented to measure the total variability in enrichment values across studies and will be used to to estimate variance due to heterogeneity. This formula was used to calculate the Q-statistic:

| [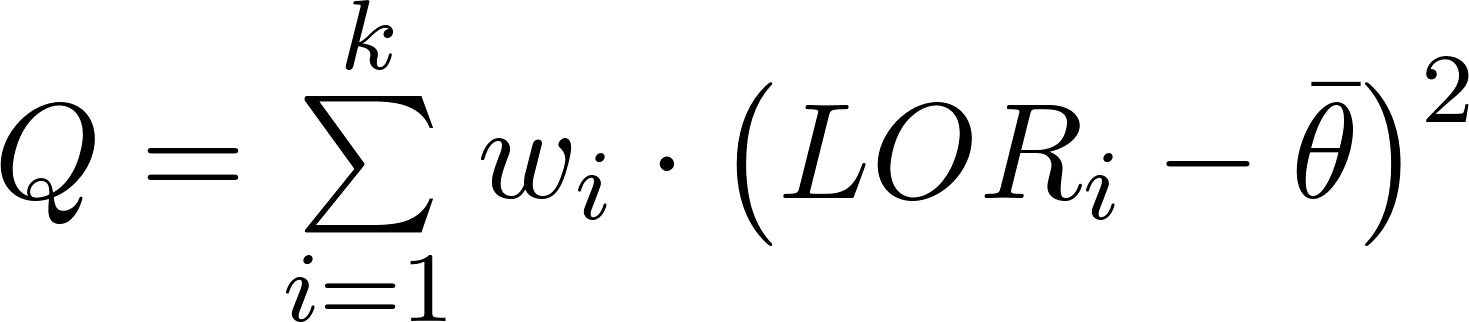](https://www.codecogs.com/eqnedit.php?latex=Q%20%3D%20%5Csum%5Climits_%7Bi%3D1%7D%5E%7Bk%7D%20w_i%20%5Ccdot%20%5Cleft(LOR_i%20-%5Cbar%7B%5Ctheta%7D%20%5Cright)%5E%7B2%7D#0) | (3) |
| --- | --- |


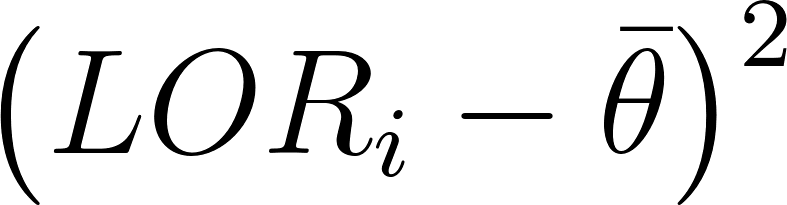


where [
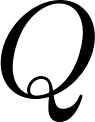
](https://www.codecogs.com/eqnedit.php?latex=Q#0) is Cochran’s Q statistic and the term represents the squared deviation of each study’s effect size from the weighted mean. A constant was computed to adjust the variance of the calculated weights and also for the estimation of the between-study variance. The calculation goes as follows:

| [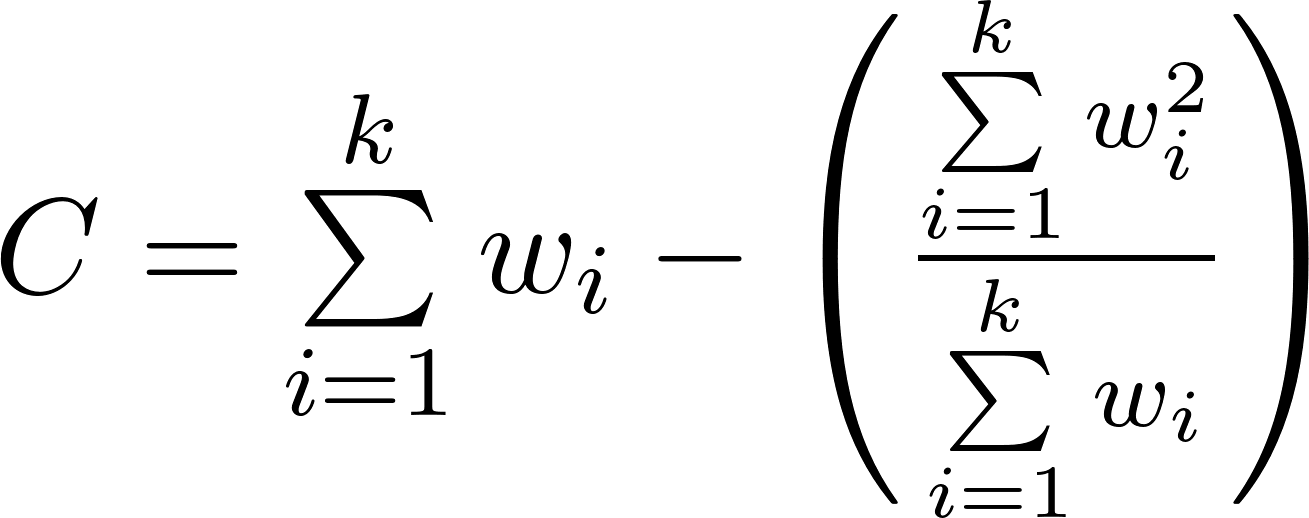](https://www.codecogs.com/eqnedit.php?latex=C%20%3D%20%5Csum%5Climits_%7Bi%3D1%7D%5E%7Bk%7Dw_i%20-%20%5Cleft(%20%5Cfrac%7B%5Csum%5Climits_%7Bi%3D1%7D%5E%7Bk%7Dw_i%5E%7B2%7D%7D%7B%5Csum%5Climits_%7Bi%3D1%7D%5E%7Bk%7Dw_i%7D%20%5Cright)#0) | (4) |
| --- | --- |

where [
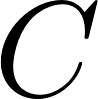
](https://www.codecogs.com/eqnedit.php?latex=C#0) is the constant, [
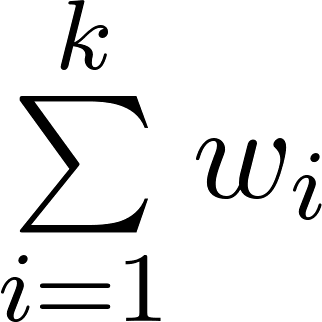
](https://www.codecogs.com/eqnedit.php?latex=%5Csum%5Climits_%7Bi%3D1%7D%5E%7Bk%7D%20w_i#0) is the sum of weights obtained from all studies and [
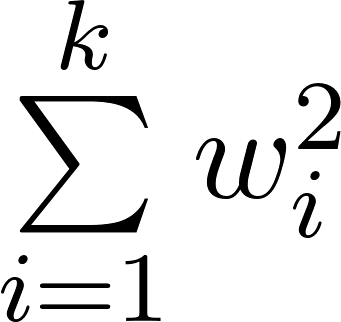
](https://www.codecogs.com/eqnedit.php?latex=%5Csum%5Climits_%7Bi%3D1%7D%5E%7Bk%7D%20w_i%5E2#0) is the sum of the squared weights. A fraction adjustment is performed, because it accounts for the variability of the weights. As implied earlier, between study-variance was estimated using the Tau-squared statistic, due to its ability to calculate the amount of variance in enrichment values due to real differences between studies rather than chance and it can ensure that the variance estimate is non-negative. The t^2^ statistic is calculated using this formula:

| [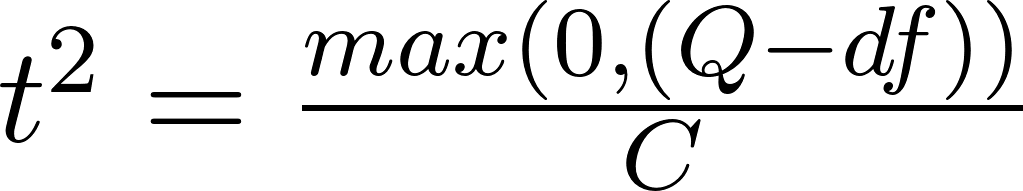](https://www.codecogs.com/eqnedit.php?latex=t%5E%7B2%7D%3D%5Cfrac%7Bmax(0%2C%20(Q%20-%20df))%7D%7BC%7D#0) | (5) |
| --- | --- |

where [
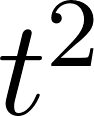
](https://www.codecogs.com/eqnedit.php?latex=t%5E2#0) is the heterogeneity variance and [
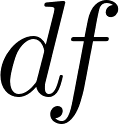
](https://www.codecogs.com/eqnedit.php?latex=df#0) represents the degrees of freedom (k-1) used to assess the statistical significance of the [
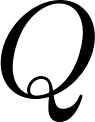
](https://www.codecogs.com/eqnedit.php?latex=Q#0) statistic against the chi-squared distribution. The final weights used for the combined enrichment values were obtained using the following random effects model:

| [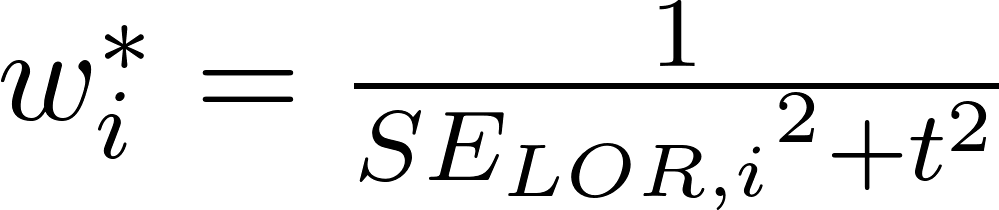](https://www.codecogs.com/eqnedit.php?latex=w_i%5E%5Cast%20%3D%20%5Cfrac%7B1%7D%7B%7BSE_%7BLOR%2Ci%7D%7D%5E2%20%2B%20t%5E2%7D#0) | (6) |
| --- | --- |

[
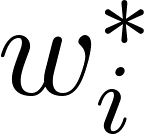
](https://www.codecogs.com/eqnedit.php?latex=w_i%5E%7B%5Cast%7D#0) is the final adjusted weight for the i^th^ study using the random effects model and[
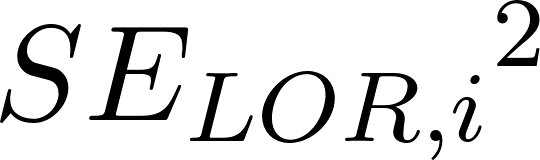
](https://www.codecogs.com/eqnedit.php?latex=%7BSE_%7BLOR%2Ci%7D%7D%5E%7B2%7D#0)is the within-study variance of the i^th^ study. The computation of the combined overall enrichment value for each item using the random effects model is represented below:

| [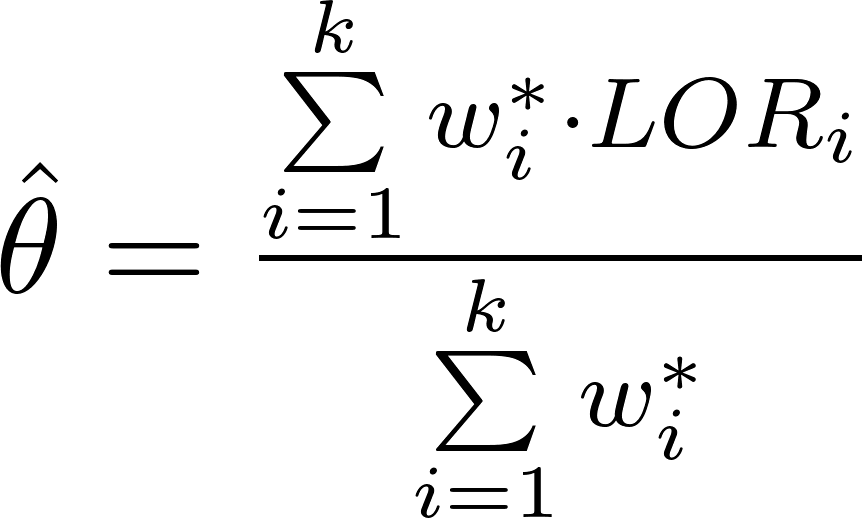](https://www.codecogs.com/eqnedit.php?latex=%5Chat%7B%5Ctheta%7D%20%3D%20%5Cfrac%7B%5Csum%5Climits_%7Bi%3D1%7D%5E%7Bk%7D%20w_i%5E%7B%5Cast%7D%20%5Ccdot%20LOR_i%20%7D%7B%5Csum%5Climits_%7Bi%3D1%7D%5E%7Bk%7D%20w_i%5E%7B%5Cast%7D%7D#0) | (7) |
| --- | --- |

[
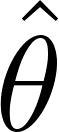
](https://www.codecogs.com/eqnedit.php?latex=%5Chat%7B%5Ctheta%7D#0) is the combined enrichment value (LOR) using the random effects model, the numerator represents the sum of adjusted LOR values while the denominator represents the sum of the adjusted weights. In all these calculations, we have assumed that taking the natural logarithm of the odds ratio provides a consistent measure of enrichment effect size. This assumption enables us to extend the enrichment analysis across species and calculate the final combined enrichment value for each item. The final step to this random-effects model was to calculate the statistical significance of the combined enrichment value against the null-hypothesis that the combined enrichment value is zero. To achieve this, first we calculated the standard error of the combined enrichment value, then we computed a modified Z-score, and with this Z-score a final p-value was obtained. The formula used for modified Z-score is the following:

| [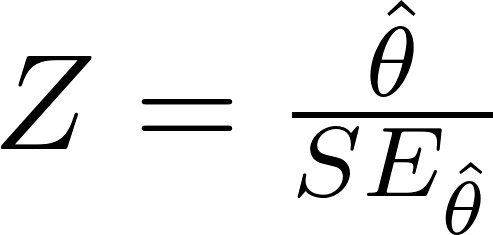](https://www.codecogs.com/eqnedit.php?latex=Z%20%3D%20%5Cfrac%7B%5Chat%7B%5Ctheta%7D%7D%7BSE_%7B%5Chat%7B%5Ctheta%7D%7D%7D#0) | (8) |
| --- | --- |

where a larger absolute value of [
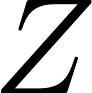
](https://www.codecogs.com/eqnedit.php?latex=Z#0) indicates a more significant deviation from the null-hypothesis and the formula for the p-value calculation:

| [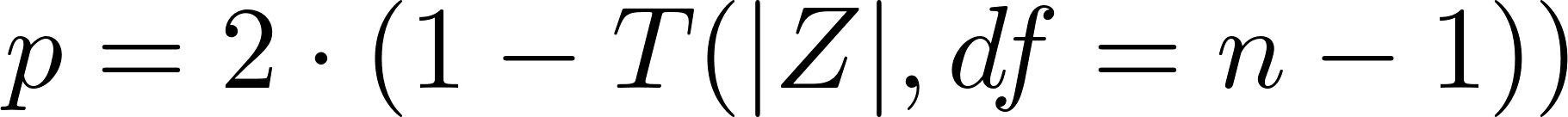](https://www.codecogs.com/eqnedit.php?latex=p%20%3D%202%20%5Ccdot(1-T(%7CZ%7C%2C%20df%20%3D%20n-1))#0) | (9) |
| --- | --- |

where [
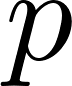
](https://www.codecogs.com/eqnedit.php?latex=p#0) represents the calculated p-value and [
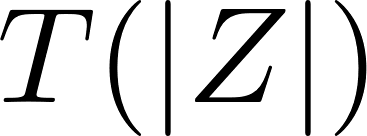
](https://www.codecogs.com/eqnedit.php?latex=T(%7CZ%7C)#0) denotes the cumulative distribution function of the t-distribution with n-1 degrees of freedom. The choice to calculate p-values based on the t-distribution was made to address the small sample size of certain underrepresented phyla. Items were filtered to keep only those with a meta-analysis adjusted p-value less than 0.05. Multiple testing correction was applied using the Benjamini-Hochberg procedure.
